# Network analysis of neuropsychiatric, cognitive, and functional complications of stroke: implications for novel treatment targets

**DOI:** 10.1111/pcn.13633

**Published:** 2024-01-29

**Authors:** Lena K.L. Oestreich, Jessica W. Lo, Maria A. Di Biase, Perminder S. Sachdev, Alice H. Mok, Paul Wright, John D. Crawford, Ben Lam, Latchezar Traykov, Sebastian Köhler, Julie E.A. Staals, Robert van Oostenbrugge, Christopher Chen, David W. Desmond, Kyung‐Ho Yu, Minwoo Lee, Aleksandra Klimkowicz‐Mrowiec, Régis Bordet, Michael J. O'Sullivan, Andrew Zalesky

**Affiliations:** ^1^ School of Psychology The University of Queensland Brisbane Queensland Australia; ^2^ Centre for Advanced Imaging and Australian Institute for Bioengineering and Nanotechnology The University of Queensland Brisbane Queensland Australia; ^3^ (CHeBA), Discipline of Psychiatry and Mental Health, School of Clinical Medicine University of New South Wales Sydney New South Wales Australia; ^4^ Melbourne Neuropsychiatry Centre, Department of Psychiatry The University of Melbourne and Melbourne Health Carlton Victoria Australia; ^5^ Department of Psychiatry Brigham and Women's Hospital, Harvard Medical School Boston Massachusetts USA; ^6^ Neuropsychiatric Institute The Prince of Wales Hospital Sydney New South Wales Australia; ^7^ Biomedical Engineering Department King's College London London UK; ^8^ Department of Neurology, UH Alexandrovska Medical University‐Sofia Sofia Bulgaria; ^9^ School for Mental Health and Neuroscience, Department of Psychiatry and Neuropsychology Maastricht University Maastricht The Netherlands; ^10^ Department of Neurology, School for Cardiovascular diseases (CARIM) Maastricht University Medical Center (MUMC+) The Netherlands; ^11^ Memory Ageing and Cognition Centre, Department of Pharmacology Yong Loo Lin School of Medicine, National University of Singapore Singapore Singapore; ^12^ Independent Researcher Florida USA; ^13^ Department of Neurology Hallym University Sacred Heart Hospital Anyang South Korea; ^14^ Department of Internal Medicine and Gerontology Jagiellonian University Medical College Krakow Poland; ^15^ Department of Pharmacology, Lille Neuroscience & Cognition University of Lille Lille France; ^16^ Department of Neurology Royal Brisbane and Women's Hospital Brisbane Queensland Australia; ^17^ Institute of Molecular Bioscience The University of Queensland Brisbane Queensland Australia; ^18^ Melbourne School of Engineering The University of Melbourne Parkville Victoria Australia

**Keywords:** affective disorders, depression, multimorbidity, stroke, worry

## Abstract

**Aim:**

Recovery from stroke is adversely affected by neuropsychiatric complications, cognitive impairment, and functional disability. Better knowledge of their mutual relationships is required to inform effective interventions. Network theory enables the conceptualization of symptoms and impairments as dynamic and mutually interacting systems. We aimed to identify interactions of poststroke complications using network analysis in diverse stroke samples.

**Methods:**

Data from 2185 patients were sourced from member studies of STROKOG (Stroke and Cognition Consortium), an international collaboration of stroke studies. Networks were generated for each cohort, whereby nodes represented neuropsychiatric symptoms, cognitive deficits, and disabilities on activities of daily living. Edges characterized associations between them. Centrality measures were used to identify hub items.

**Results:**

Across cohorts, a single network of interrelated poststroke complications emerged. Networks exhibited dissociable depression, apathy, fatigue, cognitive impairment, and functional disability modules. Worry was the most central symptom across cohorts, irrespective of the depression scale used. Items relating to activities of daily living were also highly central nodes. Follow‐up analysis in two studies revealed that individuals who worried had more densely connected networks than those free of worry (CASPER [Cognition and Affect after Stroke: Prospective Evaluation of Risks] study: *S* = 9.72, *P* = 0.038; SSS [Sydney Stroke Study]: *S* = 13.56, *P* = 0.069).

**Conclusion:**

Neuropsychiatric symptoms are highly interconnected with cognitive deficits and functional disabilities resulting from stroke. Given their central position and high level of connectedness, worry and activities of daily living have the potential to drive multimorbidity and mutual reinforcement between domains of poststroke complications. Targeting these factors early after stroke may have benefits that extend to other complications, leading to better stroke outcomes.

Stroke and major depressive disorder are two leading causes of long‐term disability and socioeconomic burden.^1^ Approximately one‐third of stroke survivors develop depression, which has adverse effects on stroke recovery: depression decreases patients' participation in rehabilitation,^2^ impairs functional recovery,^1^ and complicates a successful reintegration into the community. While antidepressants improve mood in some stroke survivors experiencing depression,^3^ they are not a curative treatment and are ineffective for many patients.

Akin to classic physical disease models, major depressive disorder and other psychiatric illnesses are assumed to meet closely defined sets of symptoms.^4^ Yet, while many individuals exhibit varying degrees and constellations of psychiatric symptoms, not all meet criteria for traditional psychiatric taxonomies. Nevertheless, many patients experience severe impairment. The most commonly reported psychiatric comorbidities of stroke include depression, apathy, fatigue, and anxiety.^5^ From a nosological perspective, depression manifests as a negative emotional state, whereas apathy is defined as a lack of emotions and motivation.^6^ Clinically, however, both syndromes share substantial symptom overlap and are often difficult to discern.^7^ Similarly, while physical and mental fatigue can occur in stroke patients in the absence of other neuropsychiatric complications, they are also common symptoms of depression and apathy.^8^

One way to better characterize psychiatric complications is to adopt a network framework to analyze the relationships among individual symptoms. Rather than considering distinct nosological entities, network analysis facilitates conceptualization of mental disorders as systems of connected symptoms that mutually influence and reinforce each other.^9^ This implies, for instance, that a person who experiences a stressful life event may worry about the reoccurrence of similar events, which may initiate a causal chain of emotions and problems: worry may lead to insomnia, to concentration problems, to feelings of worthlessness, to worry.^10^ Thus, feedback loops may create and reinforce the coevolution of symptoms.^9^ Rather than explaining the covariance between symptoms by a single common cause, network approaches consider disorders as systems that can be visualized, analyzed, and studied in their full complexities.^9^

Previous symptom network studies in major depressive disorder and poststroke depression reported that relative to patients who recover, patients with persistent depression exhibit networks with more strongly connected symptoms.^10^, ^11^ Higher network connectivity is consistent with feedback loops, whereby symptoms reinforce each other and lead to greater impairment overall.^12^ In both major depressive disorder and stroke, the symptom *sad mood* emerged as the most central symptom, meaning that it shared the most connections to other symptoms and that these connections were particularly strong relative to other connections in the network.^11^, ^13^, ^14^ While these findings indicate that treatment strategies focused on ameliorating *sad mood* could improve depression, a more comprehensive approach is needed to reduce the overall burden of stroke complications and protect against symptom cascades. In addition to their substantial comorbidity, evaluation of psychopathology in patients with stroke is further complicated by the high prevalence of cognitive deficits and varying degrees of functional disability.

Effective treatments for neuropsychiatric and nonmotor complications of stroke are not readily available. An understanding of mutual interactions between symptoms or sets of symptoms could lead to more efficient targeting of these aspects in the future. Previous network analyses have focused on ethnically homogenous patient samples. While such study designs enable better control of confounding variables, their findings are difficult to generalize and resulting treatments are likely to benefit only a small group of patients who fit the study criteria. The present study employs a network approach in multiple large and ethnically diverse stroke samples. Based on previous studies in major depression^10^, ^13^, ^14^ and poststroke depression,^11^ we hypothesized that the symptoms *sad mood* and *worry* would be the most central symptoms of poststroke depression across study sites and depression scales. Due to the complex nature of poststroke complications and their impact on recovery from stroke, we furthermore predicted that neuropsychiatric symptoms, cognitive deficits, and disabilities on activities of daily living would form one highly interrelated network as opposed to independent (separate) networks.

## Methods

### Data and contributing studies

Data were sourced from STROKOG (Stroke and Cognition Consortium), an international collaboration of observational stroke studies.^15^ Nine STROKOG studies contributed data, including five conducted in Europe,^16^, ^17^, ^18^, ^19^, ^20^ two in Asia,^21^, ^22^ one in Australia,^23^ and one in the United States.^24^ Detailed study information is provided in Table S1. For a summary of study descriptions see Dryad, https://doi.org/10.5061/dryad.m517990. All investigations were observational studies of patients admitted to hospital with stroke or transient ischemic attack (TIA). The assessment interval with the largest sample size was chosen for each study. Patients were aged >18 years and free of other neurological disorders (prior stroke was permitted). In addition to itemized depression scores, the following additional variables were obtained when available: itemized or aggregate scores on anxiety, apathy, dementia status, fatigue, other psychopathologies (i.e. Axis I disorders and symptoms), and activities of daily living. For a summary of questionnaires and neuropsychological tests, see Table S2.

Contributing studies provided evidence of informed consent obtained from participants to use and exchange data with external parties or a waiver of their local Human Research Ethics Committee (HREC). Procedures of the STROKOG consortium conform to the provisions of the Declaration of Helsinki and have been approved by the University of New South Wales HREC (HC 210709).

### Demographics, risk factors, and stroke characteristics

Demographic variables available from all studies included age, sex, ethnicity, and years of education. Cardiovascular risk factors reported for most cohorts were history of hypertension, diabetes, smoking, ischemic heart disease, atrial fibrillation, hyperlipidemia, and previous stroke. Stroke characteristics were reported as the presenting event (ischemic stroke, TIA, hemorrhagic stroke), and lesion location (left, right, bilateral, infratentorial), determined by neurological examination and confirmed by routine magnetic resonance imaging or computed tomography.

### Cognitive assessments

Cognitive scores on individual tests and, if available, standardized cognitive domain scores (memory, executive function, language, perceptual motor, attention) were included. A detailed description of the harmonization procedure for cognitive test scores is provided in Lo *et al*. (2019).^25^

### Construction of networks

For each network, missing data were handled iteratively, whereby subject rows or item columns with >30% missing data were removed in a first step.^26^ In a second step, remaining missing data were imputed using probabilistic principal component analysis. All scores were transformed into *z* scores by applying a quantile normalization. Higher scores on psychiatric symptoms signified increasingly severe psychopathology. Cognition and function‐related scores were inverted so that higher values reflected poorer cognitive performance and function. Networks were represented by symmetric M by M correlation matrices, where M denoted the number of items (i.e. nodes; shown in Table S3). The Pearson correlation coefficient between each node‐pair was computed across all patients. Correlation coefficients below a threshold of *r* < 0.2 were set to zero to remove weak correlations.^26^ Due to the large variation in neuropsychiatric, cognitive, and functional disability scales available for individual cohorts, networks were mapped separately for each study site. Additional analyses were performed by combining studies that used the Geriatric Depression Scale (GDS: Bulgarian PSS [Bulgarian Post‐Stroke Study], COAST [Cognitive Outcome After Stroke], SSS [Sydney Stroke Study], STRATEGIC [White Matter Connections and Memory: The STRATEGIC study]) and studies that used the Hamilton Depression Rating Scale (HAM‐D: EpiUSA [Epidemiologic Study of the Risk of Dementia After Stroke] and SSS) with harmonized cognitive domain scores. Items used in the final analyses and their associated node label abbreviations are presented in Tables S4–S12, S17, and S18.

### Network organization and item centrality

Graph theoretical analyses were performed in MATLAB 2020 (The MathWorks, Inc.) using the Brain Connectivity Toolbox^27^ to assess network community structure and identify the most central items, i.e. hub nodes. *Modularity* quantifies the degree to which a network is composed of groups, i.e. modules, where items within a module are more strongly correlated with each other than with items in the rest of the network. We used Newman spectral community detection algorithm with a gamma of 0.4.^28^ Hub nodes were established through three centrality measures: *betweenness* defines the amount of influence each node has on the rest of the network by determining the number of shortest paths connected to it. Nodes that lie on many shortest paths serve as bridges from one part of the network to another. *Degree* estimates how connected each node is to the rest of the network by quantifying its number of connections. *Closeness* quantifies the topological distance to all other nodes in the network by inverting the summed length of the shortest paths between a given node and all other nodes in the network. In follow‐up analyses, symptom networks were mapped separately for each of the four documented lesion locations: right hemisphere, left hemisphere, bilateral, and infratentorial. This approach allowed for an investigation into the effects of lesion location. Consistency between lesion locations was evaluated by computing the Pearson correlation coefficient for centrality measures (shown in Tables S14–S16).

## Results

After excluding participants with missing data, the total sample size was 2185 (shown in Table 1). The average age of the total sample was 66.2 years (SD = 11.5). Only female and male gender were recorded across studies, with 38% of participants identifying as female. Of the total sample, 43% were White, 43% Asian, 8% Black, 6% Hispanic, and 1% were of African, Polynesian or another ethnicity. Three cohorts included patients with ischemic stroke only (Bulgarian PSS, EpiUSA, and STRATEGIC). The remaining studies included a small number of patients with TIAs (4.4%) or hemorrhagic strokes (2%).

**Table 1:** Demographics, risk factors, and stroke characteristics by study site

	Bulgarian PSS	CASPER	COAST	EpiUSA	Hallym VCI	PROPOLIS	SSS	STRATEGIC	STROKDEM	Total
Number of participants	78	230	270	416	655	226	117	52	141	2185
Demographics										
Age, mean (SD), year	65.18 (5.73)	67.7 (11.65)	59.83 (11.44)	71.43 (7.94)	65.14 (12.12)	65.57 (12.57)	70.16 (8.58; *n* = 101)	69.47 (8.45)	63.23 (12.53)	66.21 (11.5) (*n* = 2169)
Age range, year	52–79	42–91	23–94	59–96	27–94	27–90	49–84 (*n* = 101)	51–86	25–87	23–96 (*n* = 2169)
Female gendar^a^, No. (%)	15 (19.2)	78 (33.9)	77 (28.5)	216 (51.9)	244 (37.3)	105 (46.5)	37 (36.6; *n* = 101)	13 (25)	50 (35.5)	829 (38.2; *n* = 2169)
Years of education, mean (SD)	11.4 (2.14)	N/A	7.73 (4.36)	10.16 (4.89)	9.21 (5.01)	11.98 (3.23)	10.8 (3.12)	13.94 (3.67)	11.38 (3.95)	9.98 (4.7) (*n* = 1933)
Risk factors										
Hypertension, No. (%)	66 (84.6)	169 (73.5)	194 (71.9)	301 (72.8)	373 (57.2; *n* = 652)	168 (74.3)	70 (61.9; *n* = 113)	32 (61.5)	75 (53.9)	1446 (66.4; *n* = 2177)
Diabetes, No. (%)	22 (28.2)	36 (15.7)	107 (39.6)	141 (33.9)	192 (29.4; *n* = 652)	57 (25.2)	20 (18; *n* = 111)	10 (19.2)	17 (12.1)	601 (27.6; *n* = 2176)
Smoking (current), No. (%)	22 (28.2)	170 (73.9)	113 (42; *n* = 269)	69 (16.6)	173 (26.9; *n* = 643)	58 (25.7)	58 (58.6; *n* = 99)	23 (44.2)	30 (21.3)	704 (32.8; *n* = 2147)
Ischemic heart disease^b^, No. (%)	10 (12.8)	N/A	58 (21.5)	71 (17.1; *n* = 414)	33 (5.2; *n* = 639)	31 (13.7)	25 (21.4; *n* = 112)	10 (19.2)	N/A	195 (10.9; *n* = 1791)
Atrial fibrillation, No. (%)	14 (17.9)	24 (10.5; *n* = 229)	28 (10.4)	52 (12.6; *n* = 414)	70 (10.9; *n* = 642)	34 (15)	26 (22.3; *n* = 109)	12 (23.1)	16 (11.3)	272 (12.6; *n* = 2161)
Hyperlipidemia, No. (%)	N/A	178 (77.4)	211 (78.1)	96 (23.4; *n* = 411)	225 (35; *n* = 643)	N/A	60 (51.3)	48 (92.3)	59 (41.8)	747 (40.1; *n* = 1864)
Previous stroke, No. (%)	0	16 (7)	53 (19.6)	128 (21.9)	95 (14.5)	37 (16.4)	36 (30.8)	0	13 (9.1)	203 (11.5; *n* = 1768)
Stroke characteristics										
Lesion location: right/left/bilateral/infratentorial, No. (%)	43 (55.1)/35 (44.9)/0/N/A	113 (51.4)/87 (39.5)/1 (0.5)/19 (8.6)	64 (46.4)/39 (28.3)/26 (18.8)/9 (6.5)	152 (36.5)/140 (33.7)/N/A/124 (29.8)	N/A	84 (37.8)/107 (48.2)/0/31 (14)	19 (38.8)/9 (18.4)/0/21 (42.9)	25 (48.1)/27 (51.9)/0/0	69 (49)/ 70 (49.6)/ 2 (1.4)/N/A	569 (43.2)/514 (39.1)/29 (2.2)/204 (15.5) (*n* = 1316)
Stroke type: ischemic/TIA/hemorrhagic, No. (%)	78 (100)/0/0	213 (93.4)/15 (6.6)/0	209 (77.4)/61 (22.6)/0	100 (100)/0/0	563 (99.5)/0/3 (0.5)	188 (83.2)/13 (5.8)/25 (11)	80 (79.2)/21 (20.8)/0	100 (100)/0/0	140 (99.3)/1 (0.7)/0	1938 (93.3)/96 (4.6)/44 (2.1)

*Note*. In case of missing data, sample sizes (*n*) used to calculate means and SDs or percentages are provided.

Bulgarian PSS, Bulgarian Post‐Stroke Study; CASPER, Cognition and Affect After Stroke: Prospective Evaluation of Risks; COAST, Cognitive Outcome After Stroke; EpiUSA, Epidemiologic Study of the Risk of Dementia After Stroke; Hallym VCI, Hallym Vascular Cognitive Impairment; NA, not available; PROPOLIS, Prospective Study of Pravastatin in the Elderly at Risk; SSS, Sydney Stroke Study; STRATEGIC, White Matter Connections and Memory: The STRATEGIC study; STROKDEM, Study of Factors Influencing Post‐Stroke Dementia; TIA, transient ischemic attack.

^a^ Only female and male gender were recorded.

^b^ Includes myocardial infarction.

### Network organization and item centrality by site

**Fig. 1:**
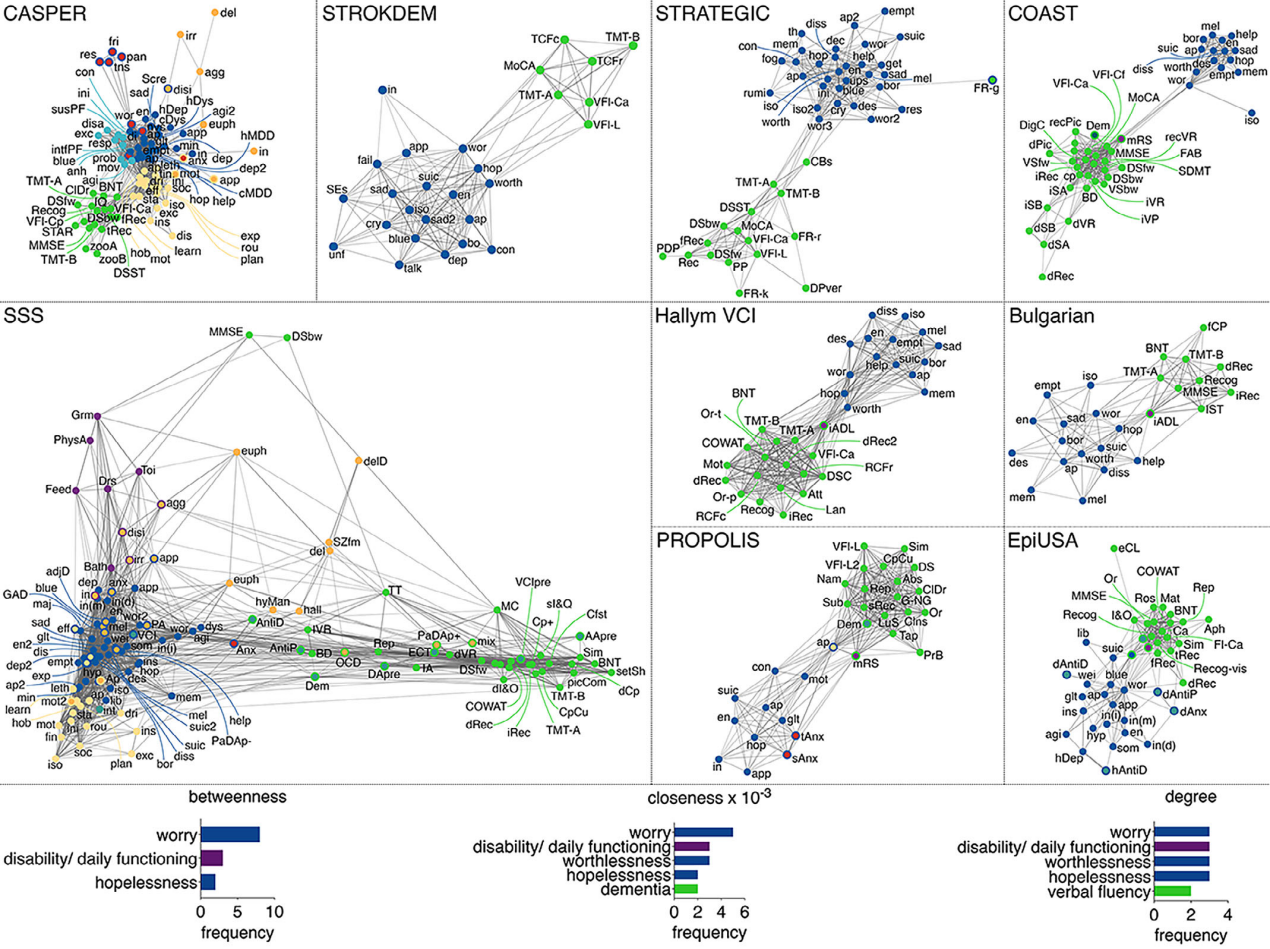
Networks by study site and most frequent centrality measures across all sites. Circles represent nodes (items/symptoms), and lines represent associations between item pairs. Thicker lines indicate stronger correlations. Circle fill represents original category and circle outline represents module category: depression (blue), cognition (green), anxiety (red), apathy (yellow), psychopathology (orange), fatigue (light blue), activities of daily living (magenta), pathology/medication (turquois). The network layout is force‐directed, whereby distant variables are weakly correlated and adjacent variables are strongly correlated. Several nodes were removed from networks due to weak correlations (*r* < 0.2) with all other network nodes. Bar graphs represent frequency of item centrality measures, whereby the relative importance of items in the overall networks was quantified by betweenness, closeness, and degree. For each parameter, the most central items across all sites are displayed. CASPER, Cognition and Affect After Stroke: Prospective Evaluation of Risks; COAST, Cognitive Outcome After Stroke; EpiUSA, Epidemiologic Study of the Risk of Dementia After Stroke; PSS, Post‐Stroke Study; SSS, Sydney Stroke Study; STRATEGIC, White Matter Connections and Memory: The STRATEGIC study; STROKDEM, Study of Factors Influencing Post‐Stroke Dementia.

For each individual cohort, all included complications generated a single network, highlighting the interrelatedness among all items (shown in Fig. 1). Networks demonstrated a modular structure, which broadly distinguished the domains of cognition, depression, apathy, fatigue, and activities of daily living. Items measuring additional pathology, psychopathology, or medication use were contained within one of those modules. Anxiety items tended to cluster with the depression modules.

Items with the highest betweenness were ‘worry’, ‘activities of daily living’, and ‘hopelessness’ (shown in Figs 1 and S1–S9). Of note, the item ‘worry’ formed bridges between all modules. Measures of degree were particularly high for the symptoms ‘worry’, ‘hopelessness’ and ‘worthlessness’. Items measuring ‘activities of daily living’ displayed similar degree estimates, closely followed by the cognitive item ‘verbal fluency’. Closeness revealed very similar patterns, with ‘worry’, ‘worthlessness’, ‘activities of daily living’, ‘hopelessness’, and ‘dementia’ exhibiting the closest connections to other nodes across networks. The most central items across studies are shown in Table S13. Lesion location did not have an effect on network configuration as evidenced by high consistency across centrality measures (see Tables S14–S16).

### Network organization and item centrality by depression scale

**Fig. 2:**
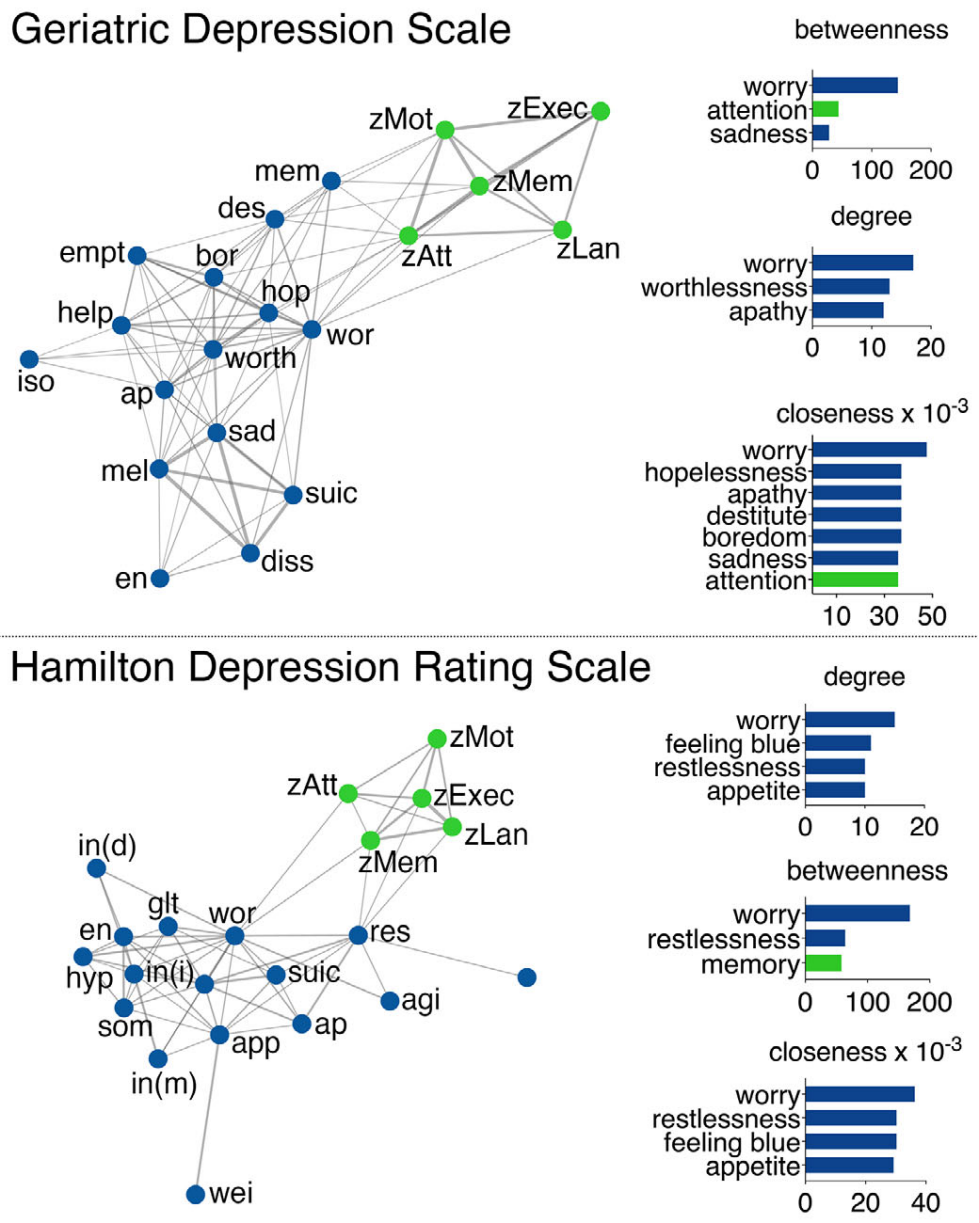
Networks by depression scale. Circles represent nodes (items/symptoms), and lines represent associations between item pairs. Thicker lines indicate stronger correlations. Modularity analysis revealed two modules for both depression scales: cognition (green) and depression (blue). Circle fill represents original category and circle outline represents module category. The network layout is force‐directed, whereby distant variables are weakly correlated and adjacent variables are strongly correlated. Bar graphs represent item centrality, whereby the relative importance of items in the overall network was quantified by betweenness, closeness, and degree. For each parameter, the top three most central items within each network are displayed.

Networks for both main measures of depression—GDS and HAM‐D—demonstrated the same large‐scale features: they were partitioned into separate depression and cognition modules (shown in Fig. 2) and the item ‘worry’ exhibited the highest betweenness, degree, and closeness (shown in Figs S10 and S11). Other prominent closeness and degree items were limited to nodes in the depression module across both depression scale networks.

### Exploratory follow‐up analysis of worry

**Fig. 3:**
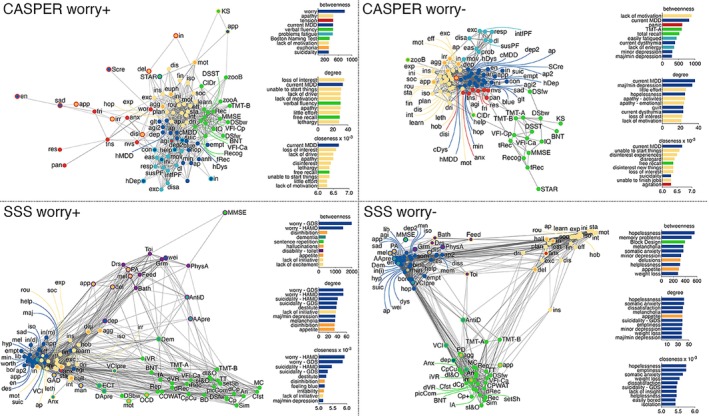
Networks by worry group. Circles represent nodes (items/symptoms), and lines represent associations between item pairs. Thicker lines indicate stronger correlations. Circle fill represents original category and circle outline represents module category: depression (blue), cognition (green), anxiety (red), apathy (yellow), psychopathology (orange), fatigue (light blue), activities of daily living (magenta), pathology/medication (turquois). The network layout is force‐directed, whereby distant variables are weakly correlated and adjacent variables are strongly correlated. Several nodes were removed from networks due to weak correlations (*r* < 0.2) with all other network nodes. Bar graphs represent item centrality, whereby the relative importance of items in the overall network was quantified by betweenness, closeness, and degree. For each parameter, the top 10 most central items within each network are displayed. CASPER, Cognition and Affect After Stroke: Prospective Evaluation of Risks; SSS, Sydney Stroke Study; worry−, patients free of symptomatic worry worry+, patients who experience symptomatic worry.

We further explored the effects of worry on network configuration in studies with the most comprehensive data, i.e. the CASPER (Colchicine After Stroke to Prevent Event Recurrence) study and SSS (Sydney Stroke Study) (shown in Table S1). To this end, we divided participants in each study into groups of patients who worried (*worry+*) and patients free of worry (*worry−*) and repeated network analysis, which yielded similar network configurations to previous analyses (shown in Figs 3 and S12–S15). The Network Comparison Test package^29^ implemented in R^30^ was used to investigate network density between groups. Networks were compared on global strength, defined as the weighted sum of the absolute connections. *Worry+* groups exhibited more connected networks than *worry−* groups, which reached significance in the CASPER study (*S* = 9.72, *P* = 0.038; *worry−* network density = 0.12, *worry+* network density = 0.23) but not in SSS (*S* = 13.56, *P* = 0.069; *worry−* network density = 0.1, *worry+* network density = 0.2).

**Fig. 4:**
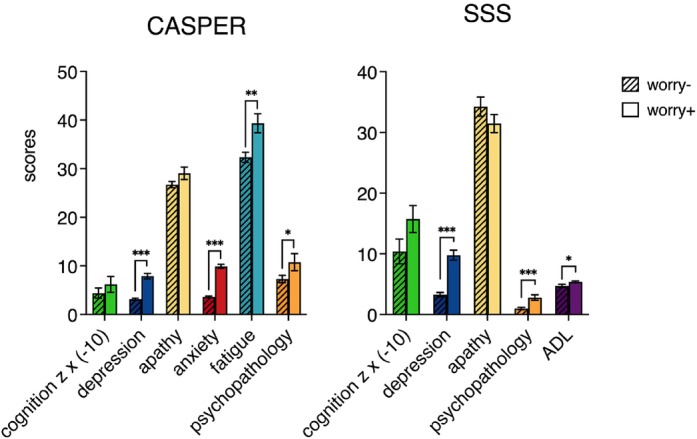
Worry group comparisons on measures of psychopathology, cognition, and functional disability. Total symptom scores, *z* scores for overall cognitive performances, and total activities of daily living (ADL) scores are plotted for worry+ and worry− groups by study site. Note that scores on cognition and ADL were inverted for ease of interpretability, with higher scores indicating greater symptom severity, poorer cognition, and more disability. CASPER, Cognition and Affect After Stroke: Prospective Evaluation of Risks; SSS, Sydney Stroke Study. *P < 0.05, **P < 0.01, ***P < 0.001.

Comparing total symptoms, cognition, and functional disability scores between groups revealed that in the CASPER study, the *worry +* group had significantly higher scores on depression (*F*(1,142) = 61.59, *P* < 0.001, $\eta_{p}^{2}$ = 0.302), anxiety (*F*(1,142) = 154.15, *P* < 0.001, $\eta_{p}^{2}$= 0.521), fatigue (*F*(1,142) = 6.44, *P* = 0.012, $\eta_{p}^{2}$ = 0.043), and psychopathology (*F*(1,142) = 4.69, *P* = 0.032, $\eta_{p}^{2}$ = 0.032) than the *worry−* group (shown in Fig. 4). In SSS, the *worry+* group had significantly higher depression (*F*(1,71) = 38.41, *P* < 0.001, $\eta_{p}^{2}$ = 0.351) and psychopathology (*F*(1,71) = 16.04, *P* < 0.001, $\eta_{p}^{2}$ = 0.184) scores and exhibited significantly more disability in daily living activities than the *worry−* group (*F*(1,71) = 5.86, *P* = 0.018, $\eta_{p}^{2}$ = 0.076).

## Discussion

In this study we used network analysis to investigate relationships among psychiatric symptoms, cognitive deficits, and functional disabilities in large and diverse stroke samples. We identified robust patterns of interrelatedness that remained consistent across different ethnic cohorts and varying assessments: individual networks of interrelated poststroke complications were characterized by partially dissociable groupings into depression, apathy, fatigue, cognition, and functional disability domains. Moreover, the symptom *worry* was the most connected item and built bridges that linked all domains. This suggests that worry plays a central role in symptomatology, linking psychiatric, cognitive, and functional complications in individuals who experienced a stroke or TIA. Further exploration of the impact of worry on network structure revealed that patients with stroke who reported worry exhibited more densely connected networks than those who were free of worry. Items relating to functional disability were also strongly connected across networks and built bridges to psychiatric symptoms and cognition. Clinically, central nodes, especially bridging nodes, can be considered transdiagnostic and treatments targeting them are likely to be effective across multiple symptoms and impairments.^14^ Consequently, our findings suggest that interventions targeted at reducing worry and functional disabilities may alleviate a wide range of poststroke complications.

Worry is a common emotional response to stroke, affecting approximately one‐quarter of survivors^31^ and worries about having additional strokes and ongoing disabilities are considered a normal part of recovery.^32^ However, if these worries persist and intensify, they may manifest as mental illness. While worry is the hallmark symptom of generalized anxiety disorder, it is also commonly observed in depressive disorders.^33^ This comorbidity was also reflected in our study, where anxiety items formed part of depression modules. In line with this finding, network studies in major depressive disorder and general anxiety disorder have reported worry to be one of the most central symptoms across both disorders.^13^, ^14^ In our study, worry also connected to other psychiatric symptoms, functional disabilities, and cognition, and was associated with poorer psychological and functional outcomes. This is clinically important insofar as it indicates that worry might be a particularly valuable target for novel interventions. Given the common occurrence of worry early after stroke, teaching stroke survivors strategies for managing their worry may prevent symptom cascades and improve cognition and disability.

Previous network studies in major depressive disorder and poststroke depression reported that less densely connected networks were related to positive prospects for recovery from depression.^10^, ^11^ Our findings indicate that alleviating worry may result in a less dense, and therefore potentially healthier, network configuration. Evidence from functional magnetic resonance imaging suggests that worry is associated with enhanced neural activity within the default mode network (DMN),^34^, ^35^ which is anchored by activity in the posterior cingulate cortex and the medial prefrontal cortex. Studies in major depressive disorder and generalized anxiety disorder found that intense and prolonged worrying is linked to excessive activation of the DMN and an inability to deactivate it during external demands,^34^, ^36^ which has also been observed in poststroke depression.^37^ Emotion Regulation Therapy, a relatively novel intervention that was specifically designed to target these neurobehavioral systems of worry,^38^ has been reported to successfully weaken the functional activity between the medial prefrontal cortex and the posterior cingulate cortex and reduce worry severity.^39^ Similarly, established therapies, such as cognitive behavioral therapy^40^ and mindfulness‐based interventions,^41^ successfully ameliorate worry in a large proportion of affected individuals. These interventions may therefore also represent valuable treatment options to stave off the negative consequences of worry on various poststroke complications.

Items related to functional disability formed central hubs that connected to psychiatric symptoms and cognitive items. Occupational therapy, especially if started early in the recovery phase has been reported to allow patients to overcome lost performance skills and safely live independently again.^42^ While the reciprocal mechanisms between functional disability, psychopathology, and cognition are not fully understood, it is possible that the initial lesion renders the brain vulnerable to the effects of prolonged periods of psychological stress caused by a loss of independence and the impact of disability, such that their collective effects trigger the development of psychiatric symptoms and lead to further cognitive deterioration. Providing access to early and ongoing occupational therapy and putting support mechanisms into place that ensure an ongoing commitment from patients to rehabilitation may therefore prevent the development of additional complications in stroke survivors.

Apathy is observed in approximately one‐third of stroke survivors. It is characterized by a lack of motivation and reduced goal‐directed behaviors.^43^ Symptoms of apathy can present behaviourally very similar to anhedonia, which is defined as diminished pleasure in previously enjoyable activities, a cardinal symptom of depression. Despite shared symptoms and a potentially similar neurobiological basis, depression and apathy are dissociable syndromes.^6^ Our findings provide support for this distinction, such that symptoms of apathy and depression formed separate symptom groups across networks. Similarly, fatigue, another common complication of stroke, shares substantial overlap with depression and apathy, but has distinct effects on stroke outcome.^44^ In our study, symptoms of fatigue were closely connected to depressive symptoms and were linked to apathy and cognition but nevertheless built an independent domain. Management of poststroke fatigue is notoriously difficult, as many pharmaceutical and behavioral interventions have limited effectiveness.^44^ Considering the substantial overlap across poststroke complications and the likelihood that connected symptoms reinforce and therefore maintain each other via feedback loops, our findings strongly advocate for a holistic approach to stroke recovery.

A clear strength of our study was that relationships among psychiatric symptoms, cognition, and functional disability were consistent across ethnically diverse stroke samples using a variety of psychopathology and cognitive assessment techniques. Importantly, these relationships remained stable regardless of lesion location. The robustness and replicability of our findings across heterogeneous stroke cohorts, testing procedures, and lesion locations means that our findings are generalizable. Nevertheless, drawing data from multiple studies might introduce unanticipated variability in sample characteristics, data acquisition, and backgrounds. Therefore, future research with larger sample sizes is essential to validate our findings. A potential constraint of network analysis lies in its sensitivity to the operationalization of symptoms, such that variations in symptom inclusion or their definitions can lead to disparate outcomes. Nonetheless, even when incorporating studies that employed heterogeneous assessment tools for depression and cognition, the consistency observed across studies and measures lends confidence in the robustness and generalizability of our findings. While the consistency of our results across stroke cohorts implies that interventions derived from the findings of this study could benefit a wide range of patients with stroke, further research into treating worry and activities of daily living are urgently needed. Another limitation is that the use of correlational measures prevented establishment of causal relationships between items. Prospective studies are required to test directional effects of poststroke complications. The present study was also limited by the fact that only one study included fatigue assessments. Conclusions about interactions with fatigue should therefore be treated with caution. Nevertheless, the close relationship between fatigue and depression is supported by evidence from previous studies.^45^

In summary, neuropsychiatric complications of stroke are highly interconnected and strongly linked to cognitive deficits and functional disability. While depression, apathy, fatigue, cognition, and functional disability clustered into dissociable domains, they also exhibited substantial interconnections. This indicates that, in order to be effective, treatments need to focus on breaking reinforcing feedback loops among poststroke complications to improve stroke outcomes. Our findings suggest that alleviating worry and promoting independence by helping stroke survivors manage disabilities represent promising avenues for future interventions that will likely be effective for a wide range of stroke complications and across ethnically diverse stroke populations.

## Disclosure statement

A.Z. is an executive member of the Queensland Neurostimulation Centre, is Editor‐in‐Chief of Neuroimage Clinical, and serves on the editorial teams of several journals. P.S. received payment for a lecture as part of the Frontiers of Psychiatry 2023 seminar, Mumbai, India, June 2023, and payment for three meetings for the Biogen Australia Medical Advisory committee in 2020 and 2021 as well as the Roche Australia Medical Advisory Committee in 2022. P.S. is also part of the executive committee of the VASCOG Society (unpaid). S.K. participated in the Scientific Advisory Board Plan Dementia Prevention Luxembourg and the Scientific Advisory Board PRECODE cohort (unpaid), served on the INTERDEM Chari Task Force Prevention, and was a member of the Project Council MOCIA consortium into lifestyle and brain health, Stakeholder Commission National Consortium of Dementia Cohorts, and Expert Advisory Panel Alzheimer Europe (all unpaid).

## Author contributions

L.O. conceptualized and designed the study, analyzed and interpreted the data, drafted the manuscript, and agrees to be accountable for all aspects of the work. A.Z. contributed to the design and interpretation of the data and revised the manuscript. M.O., J.L., M.D.B., and P.S. contributed to the interpretation of the data and revision of the manuscript. J.C. contributed to the design of the study. All other authors contributed to data acquisition and approved the version to be published.

## Supporting information


**Data S1.** Supporting Information.

## Data Availability

The data that support the findings of this study are available upon request from the STROKOG consortium (https://cheba.unsw.edu.au/consortia/strokog).
