# Supplementary material for: Network analysis of neuropsychiatric, cognitive, and functional complications of stroke: implications for novel treatment targets
Source: Psychiatry Clin Neurosci. 2024 Jan 29;78(4):229–36. doi: 10.1111/pcn.13633 (PMC11804916; doi:10.1111/pcn.13633)
Supplement: Supplementary file 1 — Data S1. Supporting Information. [file PCN-78-229-s001.pdf]

SUPPLEMENTAL MATERIAL

***SUPPLEMENTARY TABLE 1.** STUDY INFORMATION, NEUROPSYCHIATRIC AND COGNITIVE ASSESSMENT DETAILS* 2

CALCULATION OF COGNITIVE DOMAIN SCORES 4

***SUPPLEMENTARY TABLE 2.** NEUROPSYCHOLOGICAL TESTS USED TO COMPUTE DOMAIN SCORES PER STUDY* .. 5

***SUPPLEMENTARY TABLE 3.** MATRICES USED FOR SYMPTOM-FUNCTION-COGNITION NETWORKS* 6

NODE LABEL ABBREVIATIONS AND ITEM CENTRALITY MEASURES BY SITE 7

Supplementary Table 4. Bulgarian PSS: items and abbreviations 7
Supplementary Figure 1. Item centrality Bulgarian PSS. 8
Supplementary Table 5. CASPER: items and abbreviations 9
Supplementary Figure 2. Item centrality CASPER. 12
Supplementary Table 6. COAST: items and abbreviations 13
Supplementary Figure 3. Item centrality COAST. 15
Supplementary Table 7. EpiUSA: items and abbreviations 16
Supplementary Figure 4. Item centrality EpiUSA. 18
Supplementary Table 8. Hallym VCI: items and abbreviations 19
Supplementary Figure 5. Item centrality Hallym VCI. 20
Supplementary Table 9. PROPOLIS: items and abbreviations 21
Supplementary Figure 6. Item centrality PROPOLIS. 22
Supplementary Table 10. SSS: included items and abbreviations 23
Supplementary Figure 7. Item centrality SSS. 27
Supplementary Table 11. STRATEGIC: items and abbreviations 28
Supplementary Figure 8. Item centrality STRATEGIC. 30
Supplementary Table 12. STROKDEM: items and abbreviations 31
Supplementary Figure 9. Item centrality STOKDEM. 32

***SUPPLEMENTARY TABLE 13.** TOP 3 MOST CENTRAL ITEMS BY STUDY AND MEASURE* 33

***SUPPLEMENTARY TABLE 14.** SIMILARITY IN BETWEENNESS CENTRALITY ACROSS SITES* 35

***SUPPLEMENTARY TABLE 15.** SIMILARITY IN DEGREE CENTRALITY ACROSS SITES* 36

***SUPPLEMENTARY TABLE 16.** SIMILARITY IN CLOSENESS CENTRALITY ACROSS SITES* 37

Supplementary Table 17. GDS: included items and abbreviations 39
Supplementary Figure 10. Item centrality GDS. 40
Supplementary Table 18. HAM-D: included items and abbreviations 41
Supplementary Figure 11. Item centrality HAM-D. 42

CENTRALITY MEASURES: EXPLORATORY FOLLOW-UP ANALYSIS OF WORRY 43

Supplementary Figure 12. Item centrality CASPER worry+ group. 44
Supplementary Figure 13. Item centrality CASPER worry- group. 46
Supplementary Figure 14. Item centrality SSS worry+ group. 48
Supplementary Figure 15. Item centrality SSS worry- group. 50

**Supplementary Table 1.** Study information, neuropsychiatric and cognitive assessment details							
Study	Abbreviation	country	Participants with relevant data	Months: index event - assessment	Depression scales	Neuropsychiatric and disability assessments	Cognitive assessments
Bulgarian Post-Stroke Study	Bulgarian PSS	Bulgaria	78	6	GDS		MMSE; IST; immediate recall; delayed recall; recognition; TMT-A; TMT-B; BNT; figure copy
Cognition and Affect after Stroke: Prospective Evaluation of Risks	CASPER	Netherlands	230	3	HADS; MADRS	MINI; NPI; AES; FSS	MMSE; 15-Word Verbal Learning Test; digit-span forward; digit-span backward; DSST; TMT-A; TMT-B; clock drawing; cancellation; NART-R; BNT
Cognitive Outcome After Stroke	COAST	Singapore	270	3-6	GDS	mRS	MMSE; MoCA; digit-span forward; digit-span backward; visual-span forward; visual-span backward; BNT; category fluency; symbol digit modalities; digit cancellation; maze; clock drawing; block design; FAB; word list; story recall; picture recall; visual reproduction
Epidemiologic Study of the Risk of Dementia After Stroke	EpiUSA	USA	416	3	HAM-D	BFAS; Barthel Index	MMSE; recall; long-term recall; delayed recall; delayed recognition; Benton Visual Retention Test; BNT; COWAT; category fluency; repetition; comprehension; Rosen Figure Drawing Test; similarities; identities & oddities; cancellations
Hallym Vascular Cognitive Impairment	Hallym VCI	South Korea	655	3-6	GDS	iADL	MMSE; animal naming; COWAT; TMT-A; TMT-B; digit symbol coding; verbal learning test; BNT; Rey Complex Figure
PRospective Observational POLIsh Study on Post-Stroke Delirium	PROPOLIS	Poland	226	12	PHQ-9	iADL; NPI; total scores: AES, STAI, iADL	MoCA; FAB; Cognitive Test for Delirium
Sydney Stroke Study	SSS	Australia	117	36	GDS; HAM-D	NPI; SCID; AES; ADL; iADL; mRS	MMSE; logical memory; visual reproduction; digit-span forward and backward; arithmetic; mental control; BNT; TMT-A; TMT-B; symbol digit modalities; block design; figure copy; ideomotor apraxia; finger localization; stereognosis; similarities; picture completion; color sorting test; verbal fluency (animal); NART-R; COWAT; identities & oddities; set shift; sentence repetition; simple reaction time; choice reaction time
White Matter Connections and	STRATEGIC	UK	52	3-6	GDS		MoCA; immediate recall; delayed recall; recognition; TMT A; TMT B; DSST; digit-span forward; digit-span backward; letter

Memory: The STRATEGIC study							fluency; category fluency; doors and people; pyramids and palm trees; autobiographical memory; Iowa gambling task; Stroop; Corsi block; face recognition; process dissociation procedure; choice reaction time; NART-R
Study of Factors Influencing Post-Stroke Dementia	STOKDEM	France	141	6	CES-D		MoCA; TMT-A; TMT-B; Rey Complex Figure; verbal fluency (animal; letter)

Note. GDS = Geriatric Depression Scale; MMSE = mini mental state examination; IST = Isaac set test; TMT-A = trail making test Part A; TMT-B = trail making test Part B; BNT = Boston Naming Test; HADS = Hospital Anxiety and Depression Scale; MADRS = Montgomery and Asberg Depression Rating Scale; MINI = Mini International Neuropsychiatric Interview; NPI = Neuropsychiatric Inventory; AES = apathy evaluation scale; FSS = fatigue severity scale; DSST = Digit Symbol Substitution Test; NART-R = National Adult Reading Test – Revised; mRS = modified Rankin Scale; MoCA = Montreal Cognitive Assessment; FAB = Frontal Assessment Battery; HAM-D = Hamilton Depression Rating Scale; BFAS = Blessed Functional Activity Scale; COWAT = Controlled Oral Word Association Test; PHQ-9 = Patient Health Questionnaire; iADL = instrumental Activities of Daily Living scale; STAI = State-Trait Anxiety Questionnaire; SCID = Structured Clinical Interview for DSM; ADL = Activities of Daily Living scale.

Calculation of Cognitive Domain Scores

Raw neuropsychological test scores were assigned to five cognitive domains (attention, memory, language, perceptual motor, and executive function). Skewed scores were transformed to be more symmetric using a log or square transformation. Adjusted z-scores were computed for each neuropsychological test and adjusted for age, sex, and education based on the scores from a control group, using a regression model. Specifically, predicted scores in the stroke group were computed using the regression equation derived from the neurotypical comparison group. The mean z-scores computed across control groups of all tests within a domain were standardised to obtain a domain z-score. A control or normative group from the same geographical region with similar demographics to the stroke group was necessary for this standardization procedure. Cognitive domain scores could not be computed for PROPOLIS due to the absence of a comparison group and a motor performance domain score could not be computed for STRATEGIC as data were not available for this domain.

**Supplementary Table 2.** Neuropsychological tests used to compute domain scores per study					
Study	Attention	Memory	Language	Perceptual motor	Executive function
Bulgarian PSS	Trail Making Test A (1)	CERAD immediate recall; CERAD delayed recall (2)	Isaacs Set Test (1)	N/A (0)	Trail Making Test B (1)
CASPER	Trail Making Test A (1)	RAVLT (15-Word, Dutch) (1)	Verbal fluency, category (animals and professions) (1)	N/A (0)	Trail Making Test B (1)
COAST	Digit span forward; Symbol digit modalities; digit cancellation (3)	Word list test; story recall; picture recall; visual reproduction (4)	Verbal fluency (animal, food); Boston Naming Test (2)	Block design; Visual reproduction copy (2)	Digit span backward; Visual memory span backward; Maze (3)
EpiUSA	Target finding for shapes and letters (1)	Selective Reminding Test; Benton Visual Retention Test - Recognition (2)	Boston Naming Test; Verbal fluency for categories; Complex Ideation and Repetition subtests of BDAE (4)	Copying geometric figures; Benton Visual Retention Test - Matching (2)	WAIS-R Similarities subtest; Identities and Oddities; Verbal fluency for letters (3)
SSS	Trail Making Test A; Digit Span Forward; Symbol Digit Modalities Test (3)	Verbal memory recall; Logic memory; Visual memory recall; Visual reproduction (4)	Boston Naming Test; Verbal fluency (animal) (2)	Block design; Picture completion (2)	Trail Making Test B; Digit Span backward; Similarities; COWAT (4)
STRATEGIC	Trail Making Test A; Digit Span Forward; Digit Symbol Substitution (3)	FCSRT immediate recall, FCSRT delayed total recall, FCSRT total recall, FCSRT recognition (4)	Verbal fluency, category (animals and boys names) (1)	N/A (0)	Trail Making Test B; Digit Span backward; Verbal fluency, letter (3)
STOKDEM	Trail Making Test A (1)	Rey Complex Figure Test: immediate recall (1)	Verbal fluency animal (1)	Rey Complex Figure Test: Copy (1)	Trail Making Test B; Verbal fluency, letter (2)

Note. Numbers in brackets indicate the number of tests used in each domain. Bulgarian PSS = Bulgarian Post-Stroke Study; CERAD = Consortium to Establish a Registry for Alzheimer's Disease; CASPER = Cognition and Affect after Stroke: Prospective Evaluation of Risks; RAVLT = Rey Auditory Verbal Learning Test; COAST = Cognitive Outcome After Stroke; EpiUSA = Epidemiologic Study of the Risk of Dementia After Stroke; BDAE = Boston Diagnostic Aphasia Examination; WAIS-R = Wechsler Adult Intelligence Scale – Revised; SSS = Sydney Stroke Study; COWAT = Controlled Oral Word Association Test; STRATEGIC = White Matter Connections and Memory: The STRATEGIC study; FCSRT = free and cued selective reminding task; STROKDEM = Study of Factors Influencing Post-Stroke Dementia.

**Supplementary Table 3.** matrices used for symptom-function-cognition networks			
	participants x items	item pairs	correlation matrix
By site			
Bulgarian PSS	78x25	300	25x25
CASPER	230x88	3828	88x88
COAST	270x46	1035	46x46
EpiUSA	416x47	1081	47x47
Hallym VCI	655x33	528	33x33
PROPOLIS	226x33	528	33x33
SSS	117x121	7260	121x121
STRATEGIC	52x48	1128	48x48
STOKDEM	141x27	351	27x27
By depression scale			
GDS[a]	476x20	190	20x20
HAM-D[b]	533x22	231	22x22
By worry group			
SSS worry+	49x121	7260	111x111
SSS worry-	68x121	7260	105x105
CASPER worry+	177x88	3828	87x87
CASPER worry-	53x88	3828	84x84

Note. Note that the aggregation of cognitive items into domain scores and the exclusion of other items that were not recorded across all studies meant a larger number of participants reached the exclusion threshold of >30% missing data, for analyses by *depression scale,* so that sample sizes of those analyses are slightly smaller than those by *site*. Bulgarian PSS = Bulgarian Post-Stroke Study; CASPER = Cognition and Affect after Stroke: Prospective Evaluation of Risks; COAST = Cognitive Outcome After Stroke; EpiUSA = Epidemiologic Study of the Risk of Dementia After Stroke; Hallym VCI = Hallym Vascular Cognitive Impairment; PROPOLIS = Prospective Study of Pravastatin in the Elderly at Risk; SSS = Sydney Stroke Study; STRATEGIC = White Matter Connections and Memory: The STRATEGIC study; STROKDEM = Study of Factors Influencing Post-Stroke Dementia; GDS = Geriatric Depression Scale; HAM-D = Hamilton Depression Rating Scale. [a]Bulgarian PSS: n = 78; COAST: n = 245; 101 SSS: n = 101; STRATEGIC: n = 52; [b]EpiUSA: n = 416; SSS: n = 117.

Node label abbreviations and item centrality measures by site

**Supplementary Table 4.** Bulgarian PSS: items and abbreviations	
**abbreviation**	**item description**
Cognition	
MMSE	Mini Mental State Examination (total score)
TMT-A	Trail Making Test – part A
TMT-B	Trail Making Test – part B
BNT	Boston Naming Test
iRec	CERAD – immediate recall
dRec	CERAD – delayed recall
Recog	CERAD – recognition
fCp	Figure copy
IST	Isaacs set test
Depression	
diss	GDS15 – Are you basically satisfied with your life? (dissatisfied)
ap	GDS15 – Have you dropped many of your activities and interests? (apathy)
empt	GDS15 – Do you feel that your life is empty?
bor	GDS15 – Do you often get bored?
mel	GDS15 – Are you in good spirits most of the time? (melancholy)
wor	GDS15 – Are you afraid that something bad is going to happen to you? (worry)
sad	GDS15 – Do you feel happy most of the time? (sadness)
help	GDS15 – Do you often feel helpless?
iso	GDS15 – Do you prefer to stay at home rather than go out and do things? (isolation)
mem	GDS15 – Do you feel you have more problems with memory than most?
suic	GDS15 – Do you think it is wonderful to be alive now? (suicidality)
worth	GDS15 – Do you feel pretty worthless the way you are now? (worthlessness)
en	GDS15 – Do you feel full of energy? (lack of energy)
hop	GDS15 – Do you feel that your situation is hopeless?
des	GDS15 – Do you think that most people are better off than you are? (destitute)
Daily Functioning	
iADL	instrumental Activities of Daily Living – total score

Note. PSS = post-stroke study; CERAD = Consortium to Establish a Registry for Alzheimer's Disease; GDS15 = 15-item Geriatric Depression Scale.

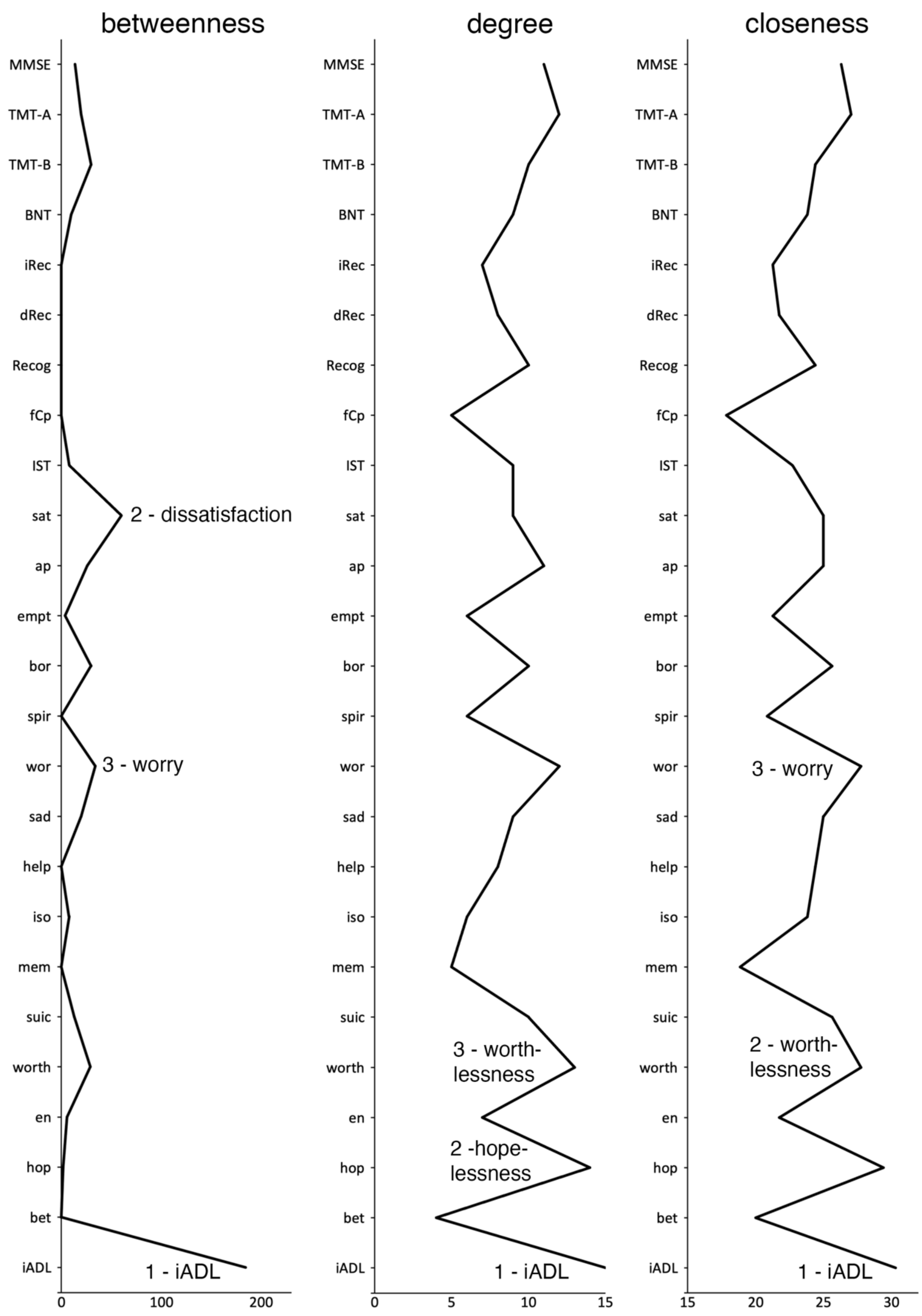

Supplementary Figure 1. Item centrality Bulgarian PSS.
The relative importance of items in the overall network was quantified by the centrality measures betweenness, degree and closeness. The top three most central items per measure are highlighted. PSS = post-stroke study. Closeness scores x 10^{-3}.

**Supplementary Table 5.** CASPER: items and abbreviations	
**abbreviation**	**item description**
Cognition	
MMSE	Mini Mental State Examination (total score)
STAR	Star cancellation
tRec	15-Word Verbal Learning Test – total recall
fRec	15-Word Verbal Learning Test – free recall
Recog	15-Word Verbal Learning Test – recognition
TMT-A	Trail Making Test – part A
TMT-B	Trail Making Test – part B
KS	BADS – key search
VFl-Ca	Verbal Fluency – category (animal)
VFl-Cp	Verbal Fluency – category (profession)
BNT	Boston Naming Test
ClDr	Clock Drawing
DSfw	Digit Span – forward
DSbw	Digit Span – backward
zooA	BADS – zoo A
zooB	BADS – zoo B
DSST	Digit Symbol Substitution Test
Depression	
hDep	History of depression
ap	HADS – I still enjoy the things I used to enjoy (apathy)
blue	HADS – I can laugh and see the funny side of things (feeling blue)
sad	HADS – I feel cheerful (sadness)
en	HADS – I feel as if I am slowed down (lack of energy)
SCre	HADS – I have lost interest in my appearance (lack of self-care)
empt	HADS – I look forward with enjoyment to things (emptiness)
anh	HADS – I can enjoy a good book or radio or TV program (anhedonia)
dep	NPI – depression (Does {S} act as if he or she is sad or in low spirits? Does he or she cry?)
hop	MADRS – Representing despondency, gloom and despair, (more than just ordinary transient low spirits) reflected in speech, facial expression, and posture (hopelessness)
help	MADRS – Representing reports of depressed mood, regardless of whether it is reflected in appearance or not. Includes low spirits, despondency or feeling being beyond help and without hope (helplessness)
agi	MADRS – Representing feelings of ill-defined discomfort, edginess, inner turmoil, mental tension mounting to either panic, dread or anguish (agitation)
in	MADRS – Representing the experience of reduced duration or depth of sleep compared to the subject's own normal pattern when well (insomnia)
app	MADRS – Representing the feeling of a loss of appetite compared with when well
con	MADRS – Representing difficulties in collecting one's thoughts mounting to incapacitating lack of concentration. Rate according to intensity, frequency, and degree of incapacity produced (difficulty concentrating)
ini	MADRS – Representing a difficulty getting started or slowness initiating and performing everyday activities (lack of initiative)
ap	MADRS – Representing the subjective experience of reduced interest in the surroundings, or activities that normally give pleasure. The ability to react with adequate emotion to circumstances or people is reduced (apathy)

glt	MADRS – Representing thoughts of guilt, inferiority, self-reproach, sinfulness, remorse and ruin (guilt)
suic	MADRS – Representing the feeling that life is not worth living, that a natural death would be welcome, suicidal thoughts, and preparations for suicide (suicidality)
cMDD	MINI – major depressive episode (current)
hMDD	MINI – major depressive episode (history)
min	MINI – minor depressive episode (two depressive symptoms for two weeks or more)
dep2	MINI – double depression (major and minor depressive episodes)
hDys	MINI – dysthymia (now persistent depressive disorder) (history)
cDys	MINI – dysthymia (now persistent depressive disorder) (current)
Anxiety	
tns	HADS – I feel tense or 'wound up' (tension)
fri	HADS – I get a sort of frightened feeling as if something awful is about to happen
wor	HADS – Worrying thoughts go through my mind
agi2	HADS – I can sit at ease and feel relaxed (agitation)
nvs	HADS – I get a sort of frightened feeling like 'butterflies' in the stomach (nervousness)
res	HADS – I feel restless as I have to be on the move
pan	HADS – I get sudden feelings of panic
anx	NPI – anxiety (Does {S} become upset when separated from you? Does he or she have any other signs of nervousness, such as shortness of breath, sighing, being unable to relax, or feeling excessively tense?)
Fatigue	
mov	FSS – My motivation is lower when I am fatigued
exc	FSS – Exercise brings on my fatigue
eas	FSS – I am easily fatigued
intfPF	FSS – Fatigue interferes with my physical functioning
prob	FSS – Fatigue causes frequent problems for me
susPF	FSS – My fatigue prevents sustained physical functioning
resp	FSS – Fatigue interferes with carrying out certain duties and responsibilities
disa	FSS – Fatigue is among my most disabling symptoms
dl	FSS – Fatigue interferes with my work, family or social life (daily living)
Other Psychopathology	
del	NPI – delusions (Does {S} believe that others are stealing from him or her, or planning to harm him or her in some way?)
hall	NPI – hallucinations (Does {S} act as if they hear voices? Do they talk to people who are not there?)
agg	NPI – agitation or aggression (Is {S} stubborn and resistive to help from others?)
euph	NPI – elation or euphoria (Does {S} appear to feel too good or act excessively happy?)
disi	NPI – disinhibition (Does {S} seem to act impulsively? For example, does the patient talk to strangers as if he or she know them, or does the patient say things that may hurt people's feelings?)
irr	NPI – irritability or lability (Is {S} impatient or cranky? Does he or she have difficulty coping with delays or waiting for planned activities?)
mot	NPI – motor disturbance (Does {S} engage in repetitive activities, such as pacing around the house, handling buttons, wrapping string, or doing other things repeatedly?)
In2	NPI – night-time behaviors or insomnia (Does {S} awaken you during the night, rise too early in the morning or take excessive naps during the day?)
app	NPI – appetite and eating (Has {S} lost or gained weight, or had a change in the food he or she likes?)
Apathy	

int	AES – I am interested in things (lack of interest)
dri	AES – I get things done during the day (lack of drive)
sta	AES – Getting things started on my own is important to me
exp	AES – I am interested in having new experiences
learn	AES – I am interested in learning new things
eff	AES – I put little effort into anything
leth	AES – I approach life with intensity (lethargy)
fin	AES – Seeing a job through to the end is important to me (finishing job/tasks)
hob	AES – I spend time doing things that interest me (no hobbies)
plan	AES – Someone has to tell me what to do each day (difficulty planning)
dis	AES – I am less concerned about my problems than I should be (disregard)
iso	AES – I have friends (isolation)
soc	AES – Getting together with friends is important to me (socializing)
exc	AES – When something good happens, I get excited
ins	AES – I have an accurate understanding of my problems (lack of insight)
rou	AES – Getting things done during the day is important to me (no routine)
ini	AES – I have initiative (lack of initiative)
mot	AES – I have motivation (lack of motivation)
ap	NPI – apathy (Does {S} seem less interested in his or her usual activities and plans of others?)

Note. CASPER = Cognition and Affect after Stroke: Prospective Evaluation of Risks; BADS = Behavioral Assessment of the Dysexecutive Syndrome; HADS = Hospital Anxiety and Depression Scale; MADRS = Montgomery and Asberg Depression Rating Scale; MINI = MINI International Neuropsychiatric Interview; FSS = Fatigue Severity Scale; NPI = Neuropsychiatric Inventory; AES = Apathy Evaluation Scale.

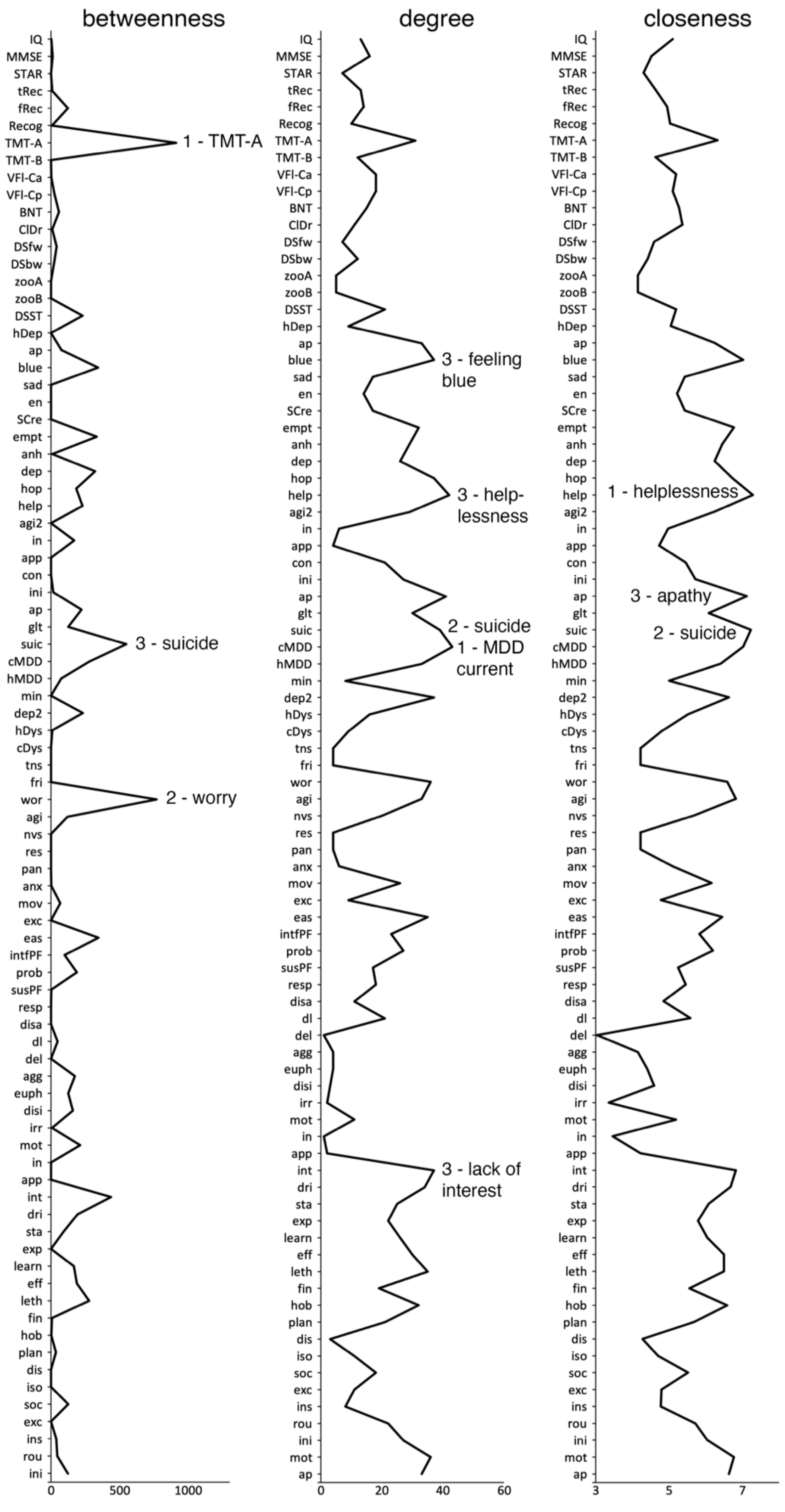

Supplementary Figure 2. Item centrality CASPER.
The relative importance of items in the overall network was quantified by the centrality measures betweenness, degree and closeness. The top three most central items per measure are highlighted. CASPER = Cognition and Affect after Stroke: Prospective Evaluation of Risks. Closeness scores x 10^{-3}. Cognitive item KS = “key search” and psychopathology item hall = “hallucinations” were removed due to weak correlations (r<0.2) with all other network nodes.

Supplementary Table 6. COAST: items and abbreviations	
**abbreviation**	**item description**
Cognition	
MMSE	Mini Mental State Examination (total score)
MoCA	Montreal Cognitive Assessment (total score)
FAB	Frontal Assessment Battery (total score)
DSfw	Digit Span – forward
DSbw	Digit Span – backward
VSfw	Visual Span – forward
VSbw	Visual Span – backward
BNT	Boston Naming Test
VFl-Ca	Verbal Fluency – category (animal)
VFl-Cf	Verbal Fluency – category (food)
iRec	immediate recall
dRec	delayed recall
Recog	recognition
iSA	Story A – immediate recall
dSA	Story A – delayed recall
iSB	Story B – immediate recall
dSB	Story B – delayed recall
iPic	WMS-r Picture – immediate recall
dPic	WMS-r Picture – delayed recall
recPic	WMS-r Picture – recognition
iVR	WMS-r Visual Reproduction – immediate recall
dVR	WMS-r Visual Reproduction – delayed recall
recVR	WMS-r Visual Reproduction – recognition
cpVR	WMS-r Visual Reproduction – copy
ClDr	Clock Drawing
BD	WAIS-r Block Design
DigC	Digit Cancellation
SDMT	Symbol Digit Modalities Test
Mz	maze
Depression	
diss	GDS15 – Are you basically satisfied with your life? (dissatisfied)
ap	GDS15 – Have you dropped many of your activities and interests? (apathy)
empt	GDS15 – Do you feel that your life is empty?
bor	GDS15 – Do you often get bored?
mel	GDS15 – Are you in good spirits most of the time? (melancholy)
wor	GDS15 – Are you afraid that something bad is going to happen to you? (worry)
sad	GDS15 – Do you feel happy most of the time? (sadness)
help	GDS15 – Do you often feel helpless?
iso	GDS15 – Do you prefer to stay at home rather than go out and do things? (isolation)
mem	GDS15 – Do you feel you have more problems with memory than most?
suic	GDS15 – Do you think it is wonderful to be alive now? (suicidality)
worth	GDS15 – Do you feel pretty worthless the way you are now?
en	GDS15 – Do you feel full of energy? (lack of energy)
hop	GDS15 – Do you feel that your situation is hopeless?
des	GDS15 – Do you think that most people are better off than you are? (destitute)
Daily Functioning	

mRS	modified Rankin Scale – degree of disability after stroke
Pathology	
Dem	Diagnosis of dementia

Note. COAST = Cognitive Outcome After Stroke; WMS-r = Wechsler Memory Scale – revised; WAIS-r = Wechsler Adult Intelligence Scale – revised; GDS15 = 15-item Geriatric Depression Scale.

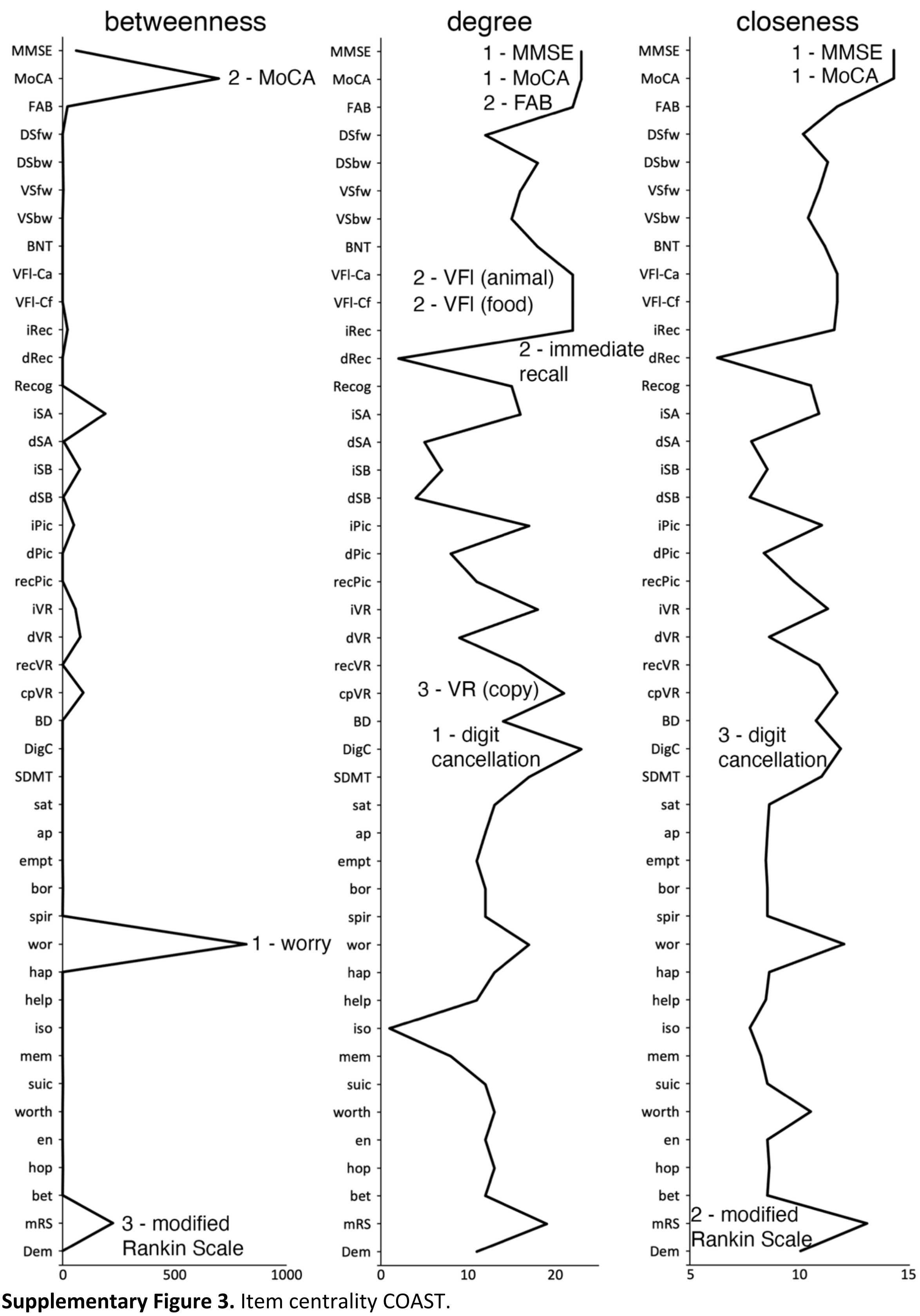

Supplementary Figure 3. Item centrality COAST.
The relative importance of items in the overall network was quantified by the centrality measures betweenness, degree and closeness. The top three most central items per measure are highlighted. COAST = Cognitive Outcome After Stroke. Closeness scores x 10^{-3}. Cognitive items ClDr = "clock drawing" and Mz = "maze" were removed due to weak correlations (r<0.2) with all other network nodes.

**Supplementary Table 7.** EpiUSA: items and abbreviations	
**abbreviation**	**item description**
Cognition	
MMSE	Mini Mental State Examination (total score)
tRec	Free and Cued Selective Reminding Test – total recall
dRec	Free and Cued Selective Reminding Test – delayed recall
fRec	Free and Cued Selective Reminding Test – free recall
Recog	Free and Cued Selective Reminding Test – recognition
Recog_vis	Benton Visual Retention Test – recognition
Or	Orientation (MMSE)
BNT	Boston Naming Test
COWAT	Phonemic fluency (COWAT)
VFl-Ca	Verbal Fluency – category (animal)
Rep	Boston Diagnostic Aphasia Examination - sentence repetition
CI	Boston Diagnostic Aphasia Examination - complex ideation
Ros	Rosen Drawing Test
Mat	Benton Visual Retention Test – matching
Sim	Similarities (WAIS-r)
I&O	Identities & oddities
hCS	Cancellation: shapes – hits
eCS	Cancellation: shapes – errors
hCL	Cancellation: letters – hits
eCL	Cancellation: letters – errors
Depression	
hDep	History of depression
blue	HAM-D – Depressed mood (gloomy attitude, pessimism about future, sadness, tendency to weep) (blue)
glt	HAM-D – Feelings of guilt
suic	HAM-D – suicide
in(i)	HAM-D – insomnia initial (difficulty falling asleep)
in(m)	HAM-D – insomnia middle (Complains of restless and disturbed during the night. Waking during night)
in(d)	HAM-D – insomnia delayed (Waking in early hours of the morning and unable to fall asleep again)
ap	HAM-D – work and interests (Slowness of thought, speech, and activity; apathy; stupor)
res	HAM-D – retardation (Restlessness associated with anxiety)
agi	HAM-D – agitation
wor	HAM-D – anxiety psychic (worry)
som	HAM-D – anxiety somatic (Gastrointestinal, Cardiovascular, Headaches, Respiratory, Genito-urinary)
app	HAM-D – somatic symptoms: gastrointestinal (Loss of appetite, abdominal heaviness; constipation)
en	HAM-D – somatic symptoms: general (Heaviness; backaches; loss of energy, fatigue) (lack of energy)
lib	HAM-D – genital symptoms (Loss of libido, menstrual disturbances)
hyp	HAM-D – hypochondriasis
wei	HAM-D – weight loss
ins	HAM-D – (lacking) insight (must be interpreted in terms of patient's understanding and background)

Other Psychopathology	
hPsy	History of psychiatric disorder
Daily Functioning	
ADL	Barthel Index (activities of daily living)
BFAS	Blessed Functional Activities Scale
Pathology and Medications	
hAntiD	History of antidepressant use
dAntiD	Antidepressants – discharge
dAntiP	Antipsychotics – discharge
dAnx	Anxiolytics – discharge
Dem	Diagnosis of dementia
Aph	Aphasia

Note. EpiUSA = Epidemiologic Study of the Risk of Dementia After Stroke; COWAT = Controlled Oral Word Association Test; WAIS-r = Wechsler Adult Intelligence Scale – revised; HAM-D = Hamilton Depression Rating Scale.

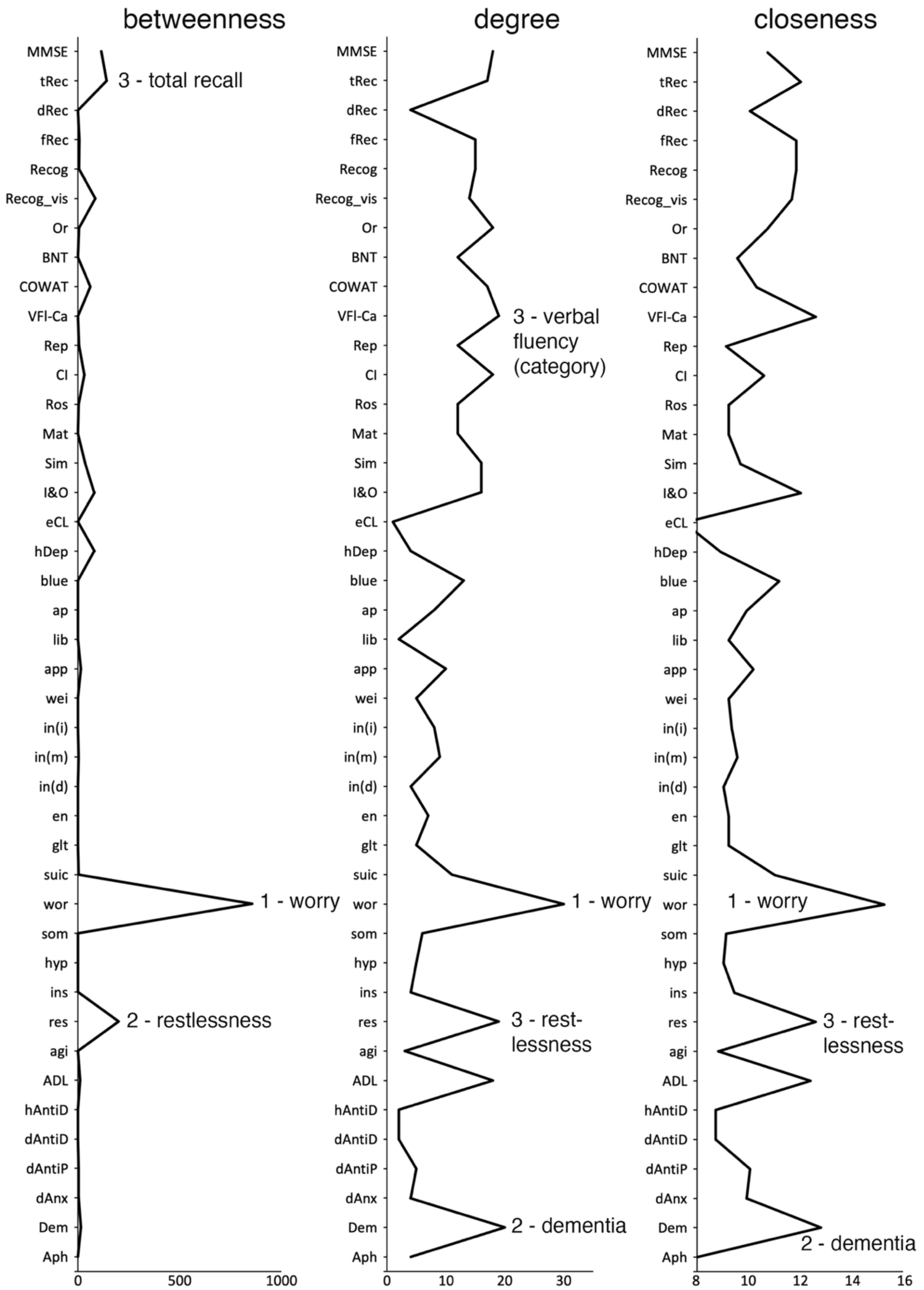

Supplementary Figure 4. Item centrality EpiUSA.
The relative importance of items in the overall network was quantified by the centrality measures betweenness, degree and closeness. The top three most central items per measure are highlighted. EpiUSA = Epidemiologic Study of the Risk of Dementia After Stroke. Closeness scores x 10^{-3}. Cognitive items hCS = "Cancellation: shapes – hits", eCS = "Cancellation: shapes – errors", hCL = "Cancellation: letters – hits", psychopathology item hPsy = "history of psychiatric disorder" and daily functioning item BFAS = "Blessed Functional Activities Scale" were removed due to weak correlations (r<0.2) with all other network nodes.

**Supplementary Table 8.** Hallym VCI: items and abbreviations	
**abbreviation**	**item description**
Cognition	
Or-t	Orientation – time (MMSE)
Or-p	Orientation – place (MMSE)
iRec	Immediate recall (MMSE)
Att	Attention (MMSE)
dRec	Delayed Recall (MMSE)
Lan	Language (MMSE)
Mot	Motor performance/ perceptual (MMSE)
VFl-Ca	Verbal Fluency – category (animal)
COWAT	Phonemic fluency (COWAT)
DSC	Digit symbol coding
TMT-A	Trail Making Test – part A
TMT-B	Trail Making Test – part B
BNT	Boston Naming Test
RCFc	Rey Complex Figure Test – copy
RCFr	Rey Complex Figure Test – recall
dRec2	Verbal Learning Test – delayed recall
Recog	Verbal Learning Test – recognition
Depression	
diss	GDS15 – Are you basically satisfied with your life? (dissatisfied)
ap	GDS15 – Have you dropped many of your activities and interests? (apathy)
empt	GDS15 – Do you feel that your life is empty?
bor	GDS15 – Do you often get bored?
mel	GDS15 – Are you in good spirits most of the time? (melancholy)
wor	GDS15 – Are you afraid that something bad is going to happen to you? (worry)
sad	GDS15 – Do you feel happy most of the time? (sadness)
help	GDS15 – Do you often feel helpless?
iso	GDS15 – Do you prefer to stay at home rather than go out and do things? (isolation)
mem	GDS15 – Do you feel you have more problems with memory than most?
suic	GDS15 – Do you think it is wonderful to be alive now? (suicidality)
worth	GDS15 – Do you feel pretty worthless the way you are now?
en	GDS15 – Do you feel full of energy? (lack of energy)
hop	GDS15 – Do you feel that your situation is hopeless?
des	GDS15 – Do you think that most people are better off than you are? (destitute)
Daily Functioning	
iADL	instrumental Activities of Daily Living – total score

Note. VCI = Vascular Cognitive Impairment; MMSE = Mini Mental State Examination; MoCA = Montreal Cognitive Assessment; COWAT = Controlled Oral Word Association Test; GDS15 = 15-item Geriatric Depression Scale.

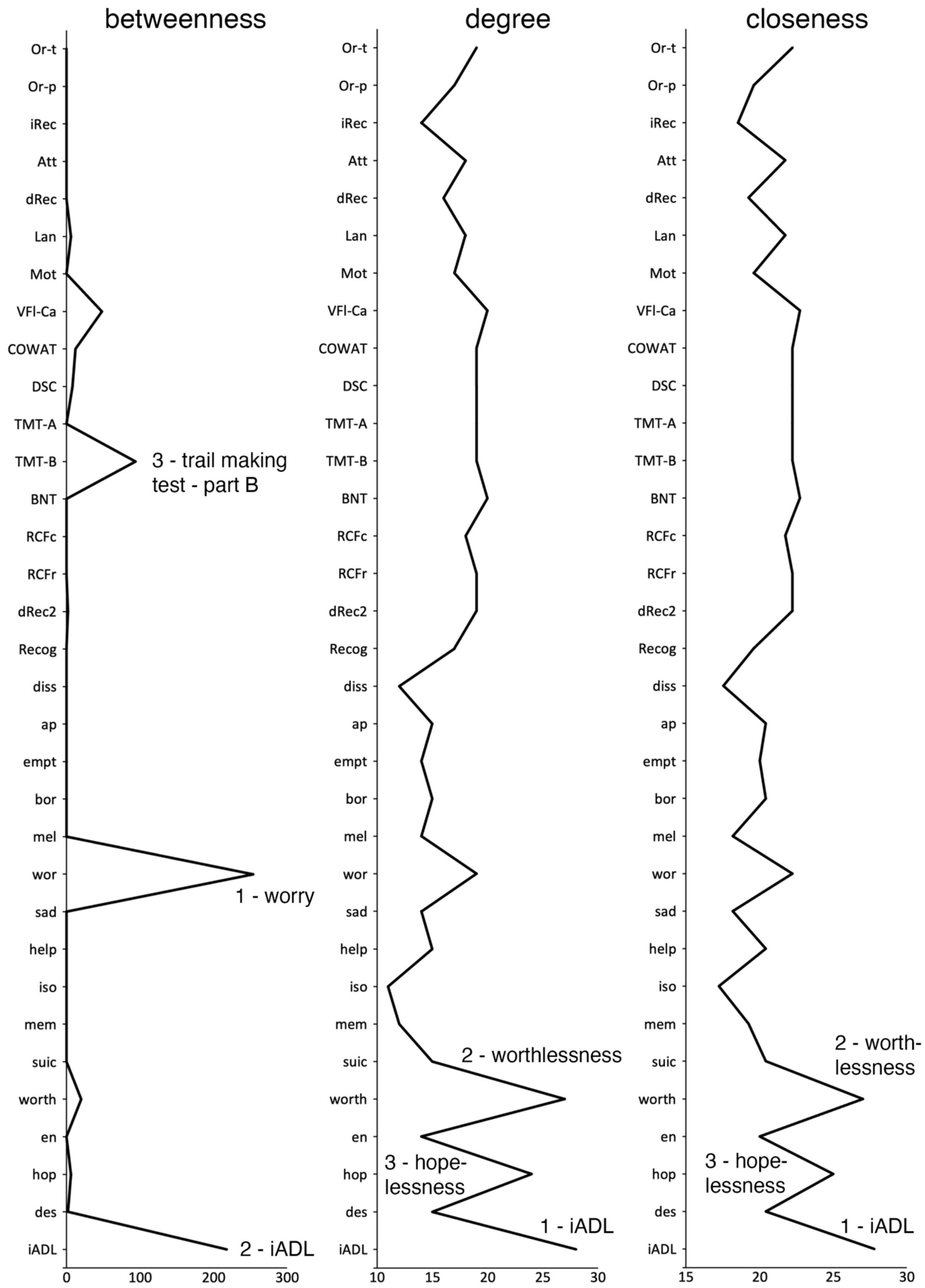

Supplementary Figure 5. Item centrality Hallym VCI.
The relative importance of items in the overall network was quantified by the centrality measures betweenness, degree and closeness. The top three most central items per measure are highlighted. VCI = Vascular Cognitive Impairment. Closeness scores x 10^{-3}.

**Supplementary Table 9.** PROPOLIS: items and abbreviations	
**abbreviation**	**item description**
Cognition	
TMT-B	Trail Making Test – part B (MoCA)
CpCu	Copy cube
ClDr	Logical Memory I – immediate recall
Nam	Logical Memory II – delayed recall
DS	Digit Span – forward and backward combined (MoCA)
Tap	Tapping (MoCA)
Sub	Subtraction (MoCA)
Rep	Repetition (MoCA)
VFl-L	Verbal Fluency – letter (MoCA)
Abs	Abstraction (MoCA)
dRec	Delayed Recall (MoCA)
Or	Orientation (MoCA)
Sim	Similarities (FAB)
VFl-L2	Verbal Fluency – letter (FAB)
LuS	Luria Sequence (FAB)
CIns	Conflicting Instructions (FAB)
G-NG	Go – No Go (FAB)
PrB	Prehension Behavior (FAB)
Depression	
ap	PHQ-9 – little interest or pleasure in doing things (apathy)
hop	PHQ-9 – feeling down, depressed, or hopeless
in	PHQ-9 – trouble falling or staying asleep, or sleeping too much (insomnia)
en	PHQ-9 – feeling tired or having little energy (lack of energy)
app	PHQ-9 – Poor appetite or overeating
glt	PHQ-9 – Feeling bad about yourself – or that you are a failure or have let yourself or your family down (guilt)
con	PHQ-9 – trouble concentrating on things, such as reading the newspaper or watching television (difficulty concentrating)
mot	PHQ-9 – Moving or speaking so slowly that other people could have noticed. Or the opposite – being so fidgety or restless that you have been moving around a lot more than usual (motor disturbance)
suic	PHQ-9 – thoughts that you would be better off dead, or of hurting yourself (suicidality)
Apathy	
ap	Apathy Evaluation Scale – total score
Anxiety	
sAnx	State Trait Anxiety Inventory – state
tAnx	State Trait Anxiety Inventory – trait
Daily Functioning	
mRS	modified Rankin Scale – degree of disability after stroke
iADL	instrumental Activities of Daily Living – total score
Pathology	
Dem	Diagnosis of dementia

Note. PROPOLIS = Prospective Study of Pravastatin in the Elderly at Risk; MoCA = Montreal Cognitive Assessment; FAB = Frontal Assessment Battery; PHQ-9 = Patient Health Questionnaire.

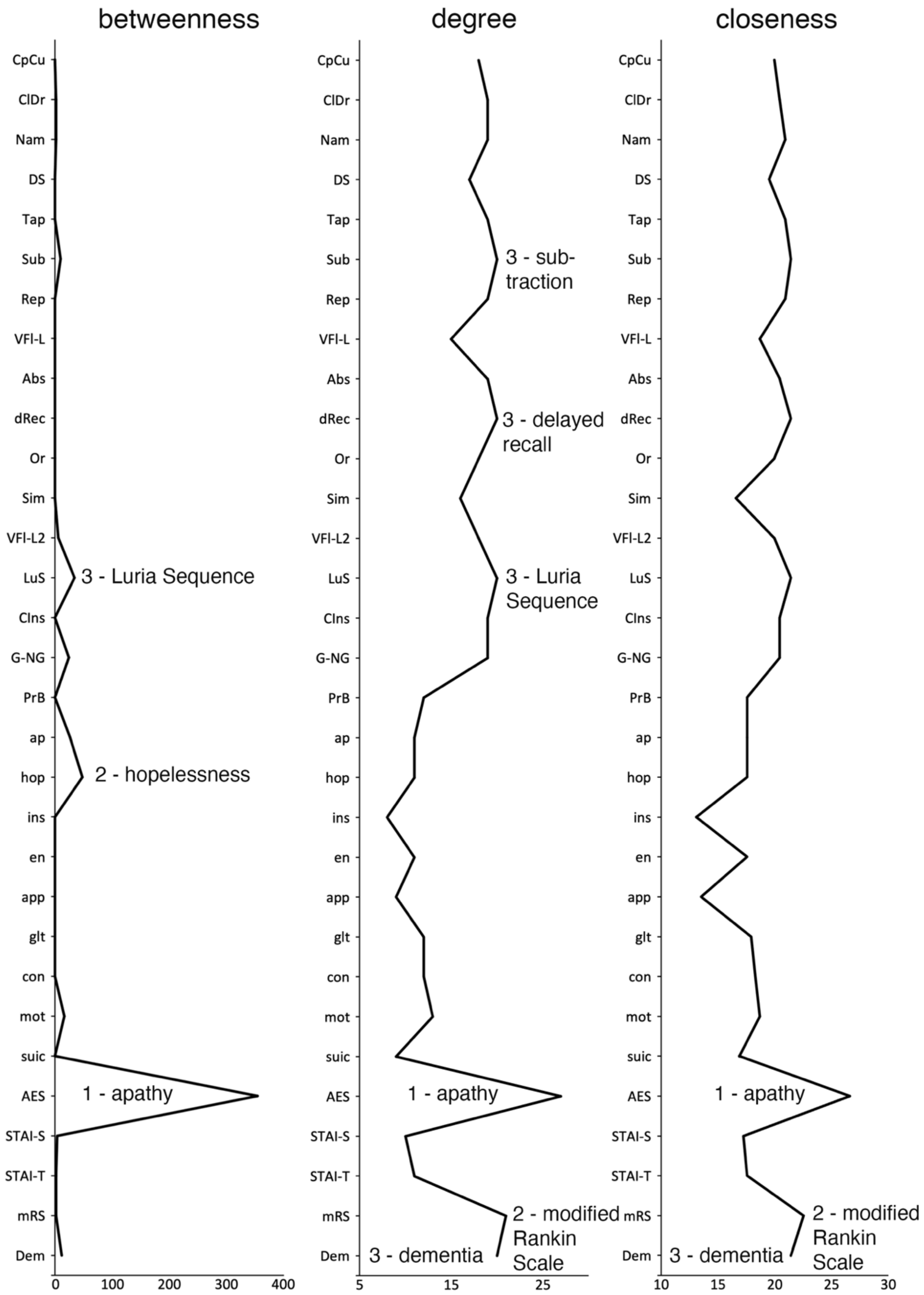

Supplementary Figure 6. Item centrality PROPOLIS.
The relative importance of items in the overall network was quantified by the centrality measures betweenness, degree and closeness. The top three most central items per measure are highlighted. VCI = Vascular Cognitive Impairment. PROPOLIS = Prospective Study of Pravastatin in the Elderly at Risk. Closeness scores x 10^{-3}. Cognitive item CpCu = "copy cube", and daily functioning item iADl = "instrumental Activities of Daily Living – total score" were removed due to weak correlations (r<0.2) with all other network nodes.

**Supplementary Table 10.** SSS: included items and abbreviations	
**abbreviation**	**item description**
Cognition	
MMSE	Mini Mental State Examination (total score)
MC	Mental control
iRec	Immediate recall
dRec	Delayed recall
iVR	Visual Reproduction – immediate recall
dVR	Visual Reproduction – delayed recall
DSfw	Digit Span – forward
DSbw	Digit Span – backward
picCom	Picture completion
BD	Block Design
Ari	Arithmetic
Sim	Similarities
TMT-A	Trail Making Test – part A
TMT-B	Trail Making Test – part B
Cp+	Copy cross
CpCu	Copy cube
dCp	Draw copy 1 + 2
Cfst	Color from sort test
TT	Token Test
IA	Ideomotor Apraxia
BNT	Boston Naming Test
COWAT	Phonemic fluency (COWAT)
VFl-Ca	Verbal Fluency – category (animal)
sI&O	Identities & oddities (same)
dI&O	Identities & oddities (different)
setSh	set shift
Rep	Repetition (sentence)
Depression	
blue	HAM-D – Depressed mood (gloomy attitude, pessimism about the future, feeling of sadness, tendency to weep) (blue)
glt	HAM-D – Feelings of guilt
suic	HAM-D – suicide
in(i)	HAM-D – insomnia initial (difficulty falling asleep)
in(m)	HAM-D – insomnia middle (Complains of being restless and disturbed during the night. Waking during the night)
in(d)	HAM-D – insomnia delayed (Waking in early hours of morning & unable to fall asleep again)
ap	HAM-D – work and interests (Slowness of thought, speech, and activity; apathy; stupor)
res	HAM-D – retardation (Restlessness associated with anxiety)
agi	HAM-D – agitation
wor	HAM-D – anxiety psychic (worry)
som	HAM-D – anxiety somatic (Gastrointestinal, indigestion, Cardiovascular, palpitation, Headaches, Respiratory, Genito-urinary, etc.)
app	HAM-D – somatic symptoms: gastrointestinal (Loss of appetite, heavy feeling in abdomen; constipation)
en	HAM-D – somatic symptoms: general (Heaviness in limbs, back or head; diffuse backache; loss of energy and fatiguability) (lack of energy)

lib	HAM-D – genital symptoms (Loss of libido, menstrual disturbances)
hyp	HAM-D – hypochondriasis
wei	HAM-D – weight loss
ins	HAM-D – insight (Insight must be interpreted in terms of patient's understanding and background.) (lack of insight)
diss	GDS15 – Are you basically satisfied with your life? (dissatisfied)
ap2	GDS15 – Have you dropped many of your activities and interests? (apathy)
empt	GDS15 – Do you feel that your life is empty?
bor	GDS15 – Do you often get bored?
mel	GDS15 – Are you in good spirits most of the time? (melancholy)
wor2	GDS15 – Are you afraid that something bad is going to happen to you? (worry)
sad	GDS15 – Do you feel happy most of the time? (sadness)
help	GDS15 – Do you often feel helpless?
iso	GDS15 – Do you prefer to stay at home rather than go out and do things? (isolation)
mem	GDS15 – Do you feel you have more problems with memory than most?
suic2	GDS15 – Do you think it is wonderful to be alive now? (suicidality)
worth	GDS15 – Do you feel pretty worthless the way you are now?
en2	GDS15 – Do you feel full of energy? (lack of energy)
hop	GDS15 – Do you feel that your situation is hopeless?
des	GDS15 – Do you think that most people are better off than you are? (destitute)
dep	NPI – depression (Does {S} act as if he or she is sad or in low spirits? Does he or she cry?)
dys	SCID – dysthymia (now persistent depressive disorder)
dep2	SCID – double depression (major depressive episode superimposed on antecedent dysthymia)
maj	SCID – major depressive episode
min	SCID – minor depressive episode (two depressive symptoms for two weeks or more)
Apathy	
int	AES – I am interested in things (lack of interest)
dri	AES – I get things done during the day (lack of drive)
sta	AES – Getting things started on my own is important to me
exp	AES – I am interested in having new experiences
learn	AES – I am interested in learning new things
eff	AES – I put little effort into anything
leth	AES – I approach life with intensity (lethargy)
fin	AES – Seeing a job through to the end is important to me (finishing job/tasks)
hob	AES – I spend time doing things that interest me (no hobbies)
plan	AES – Someone has to tell me what to do each day (difficulty planning)
dis	AES – I am less concerned about my problems than I should be (disregard)
iso	AES – I have friends (isolation)
soc	AES – Getting together with friends is important to me (socializing)
exc	AES – When something good happens, I get excited
ins	AES – I have an accurate understanding of my problems (lack of insight)
rou	AES – Getting things done during the day is important to me (no routine)
ini	AES – I have initiative (lack of initiative)
mot	AES – I have motivation (lack of motivation)
ap	NPI – apathy (Does {S} seem less interested in his/ her usual activities and plans of others?)
Other Psychopathology	
del	NPI – delusions (Does {S} believe that others are stealing from him or her, or planning to harm him or her in some way?)

hall	NPI – hallucinations (Does {S} act as if he or she hears voices? Does he or she talk to people who are not there?)
agg	NPI – agitation or aggression (Is {S} stubborn and resistive to help from others?)
anx	NPI – anxiety (Does {S} become upset when separated from you? Does he or she have any other signs of nervousness, such as shortness of breath, sighing, being unable to relax, or feeling excessively tense?)
euph	NPI – elation or euphoria (Does {S} appear to feel too good or act excessively happy?)
disi	NPI – disinhibition (Does {S} seem to act impulsively? For example, does the patient talk to strangers as if he or she know them, or does {S} say things that may hurt people's feelings?)
irr	NPI – irritability or lability (Is {S} impatient or cranky? Does he or she have difficulty coping with delays or waiting for planned activities?)
mot	NPI – motor disturbance (Does {S} engage in repetitive activities, such as pacing around the house, handling buttons, wrapping string, or doing other things repeatedly?)
in	NPI – night-time behaviors or insomnia (Does {S} awaken you during the night, rise too early in the morning or take excessive naps during the day?)
app	NPI – appetite and eating (Has {S} lost or gained weight, or had a change in the food he or she likes?)
SZ	SCID – schizophrenia
SZfm	SCID – schizophreniform disorder
delD	SCID – delusional disorder
bPD	SCID – brief psychotic disorder
mPD	SCID – medication induced psychotic disorder
sPsy	SCID – substance induced psychotic disorder
oPsy	SCID – psychotic disorder not otherwise specified
mel	SCID – melancholia
man	SCID – manic episode
mix	SCID – mood disorder with mixed features
hyMan	SCID – hypomania
adjD	SCID – adjustment disorder
PA	SCID – panic attacks
Ap	SCID – agoraphobia
PaDAp+	SCID – panic disorder with agoraphobia
PaDAp-	SCID – panic disorder without agoraphobia
GAD	SCID – generalized anxiety disorder
OCD	SCID – obsessive compulsive disorder
Daily Functioning	
Toi	ADL – Toilet
Feed	ADL – Feeding
Drs	ADL – Dressing
Grm	ADL – Grooming (neatness, hair, nails, hands, face, clothing)
PhysA	ADL – Physical Ambulation
Bath	ADL – Bathing
Pathology and Medications	
AntiD	Antidepressants – baseline
Anx	Anxiolytics – baseline
AntiP	Antipsychotics – baseline
ECT	Electroconvulsive therapy – baseline
Dem	Diagnosis of dementia
VCIpre	Vascular Cognitive Impairment prior to stroke
VCI	Vascular Cognitive Impairment

AApre	Alcohol abuse prior to stroke
DApre	Drug abuse prior to stroke

Note. SSS = Sydney Stroke Study; COWAT = Controlled Oral Word Association Test; HAM-D = Hamilton Depression Rating Scale; GDS15 = 15-item Geriatric Depression Scale; NPI = Neuropsychiatric Inventory; {S} = subject; SCID = Structured Clinical Interview for DSM-IV; AES = Apathy Evaluation Scale; ADL = Activities of Daily Living (physical self-maintenance scale).

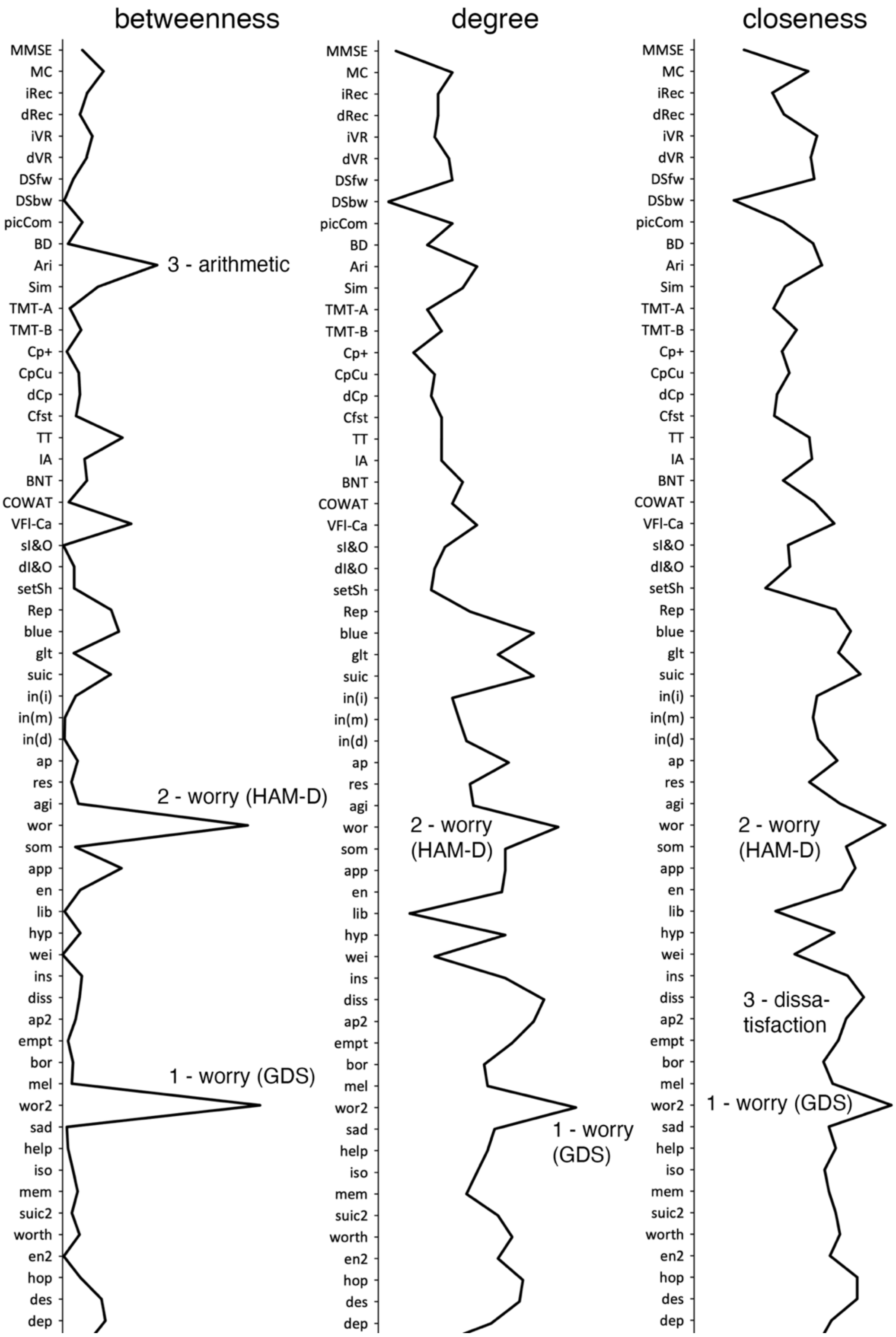

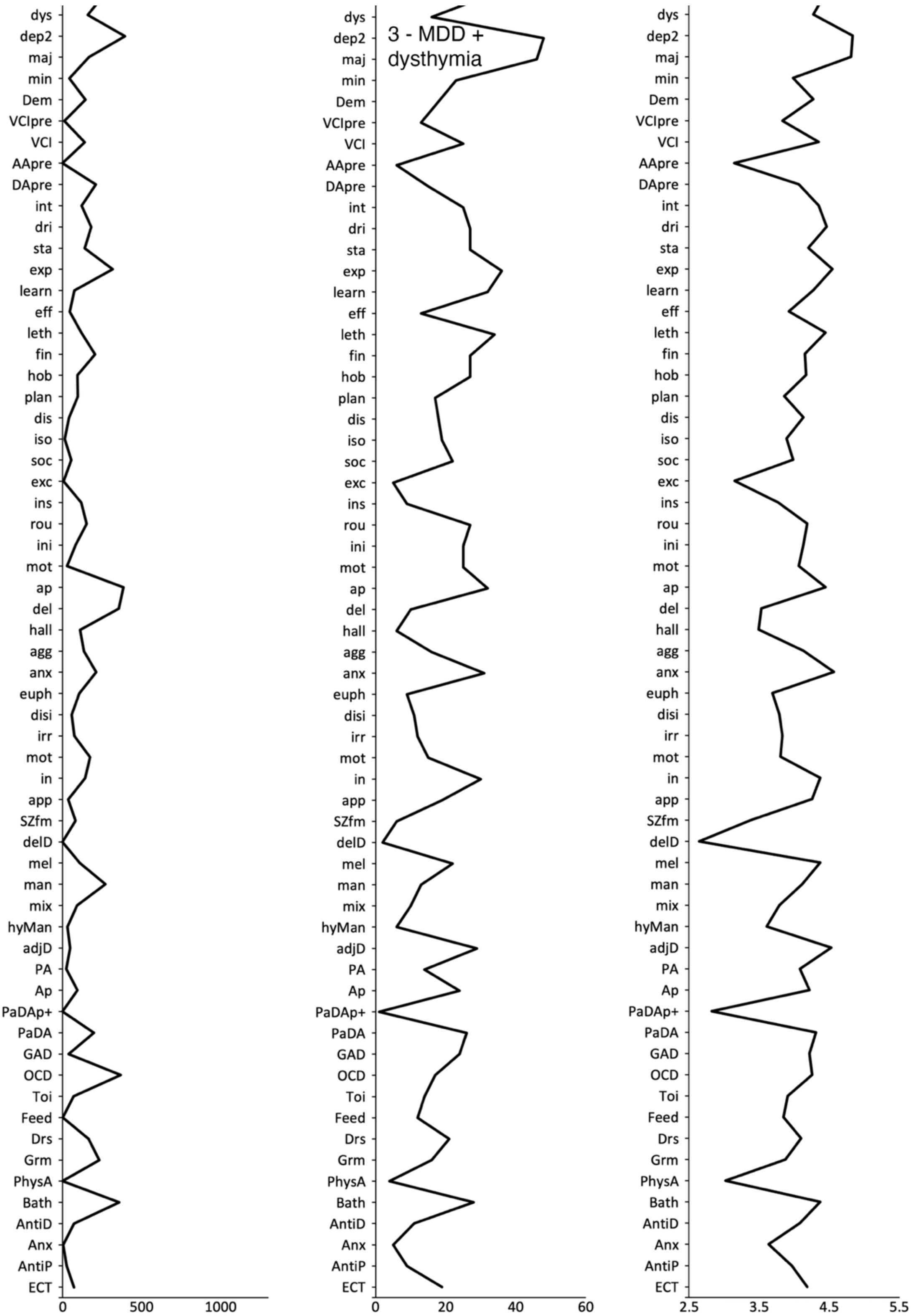

Supplementary Figure 7. Item centrality SSS.
The relative importance of items in the overall network was quantified by the centrality measures betweenness, degree and closeness. The top three most central items per measure are highlighted. VCI = Vascular Cognitive Impairment. SSS = Sydney Stroke Study. Closeness scores x 10^{-3}.
Psychopathology items SZ = “schizophrenia”, bPD = “brief psychotic disorder”, mPD = “medication induced psychotic disorder”, sPsy = “substance induced psychotic disorder” and oPsy = “psychotic disorder not otherwise specified” were removed due to weak correlations (r<0.2) with all other network nodes.

**Supplementary Table 11.** STRATEGIC: items and abbreviations	
**abbreviation**	**item description**
Cognition	
MoCA	Montreal Cognitive Assessment (total score)
fRec	Free and Cued Selective Reminding Test – free recall
tRec	Free and Cued Selective Reminding Test – total recall
TMT-A	Trail Making Test – part A
TMT-B	Trail Making Test – part B
DSST	Digit Symbol Substitution Test
DSfw	Digit Span – forward
DSbw	Digit Span – backward
VFl-L	Verbal Fluency – letter
VFl-Ca	Verbal Fluency – category (animal)
PP	Pyramids and Palm trees
DPver	Doors and People – verbal recall
DPvis	Doors and People – visual recall
PDP	Process Dissociation Procedure – recollection
FR-r	Face Recognition – remember
FR-k	Face Recognition – know
FR-g	Face Recognition – guess
CBs	Corsi Block test – span
Depression	
diss	GDS30 – Are you basically satisfied with your life? (dissatisfied)
ap	GDS30 – Have you dropped many of your activities and interests? (apathy)
empt	GDS30 – Do you feel that your life is empty?
bor	GDS30 – Do you often get bored?
wor	GDS30 – Are you hopeful about the future? (worry)
th	GDS30 – Are you bothered by thoughts you can't get out of your head?
mel	GDS30 – Are you in good spirits most of the time? (melancholia)
wor2	GDS30 – Are you afraid that something bad is going to happen to you? (worry)
sad	GDS30 – Do you feel happy most of the time? (sadness)
help	GDS30 – Do you often feel helpless?
res	GDS30 – Do you often get restless and fidgety?
iso	GDS30 – Do you prefer to stay at home rather than go out and do things? (isolation)
wor3	GDS30 – Do you frequently worry about the future? (worry)
mem	GDS30 – Do you feel you have more problems with memory than most?
suic	GDS30 – Do you think it is wonderful to be alive now? (suicidality)
blue	GDS30 – Do you feel downhearted and blue?
worth	GDS30 – Do you feel pretty worthless the way you are now?
rumi	GDS30 – Do you worry a lot about the past? (rumination)
ap2	GDS30 – Do you find life very exciting? (apathy)
ini	GDS30 – Is it hard for you to get started on new projects? (lack of initiative)
en	GDS30 – Do you feel full of energy? (lack of energy)
hop	GDS30 – Do you feel that your situation is hopeless?
des	GDS30 – Do you think that most people are better off than you are? (destitute)
ups	GDS30 – Do you frequently get upset over little things?
cry	GDS30 – Do you frequently feel like crying?
con	GDS30 – Do you have trouble concentrating?
get	GDS30 – Do you enjoy getting up in the morning? (difficulty getting up)

iso2	GDS30 – Do you prefer to avoid social occasions? (isolation)
dec	GDS30 – Is it easy for you to make decisions? (difficulty making decisions)
fog	GDS30 – Is your mind as clear as it used to be? (brain fog)

Note. STRATEGIC = White Matter Connections and Memory: The STRATEGIC study; GDS30 = 30-item Geriatric Depression Scale.

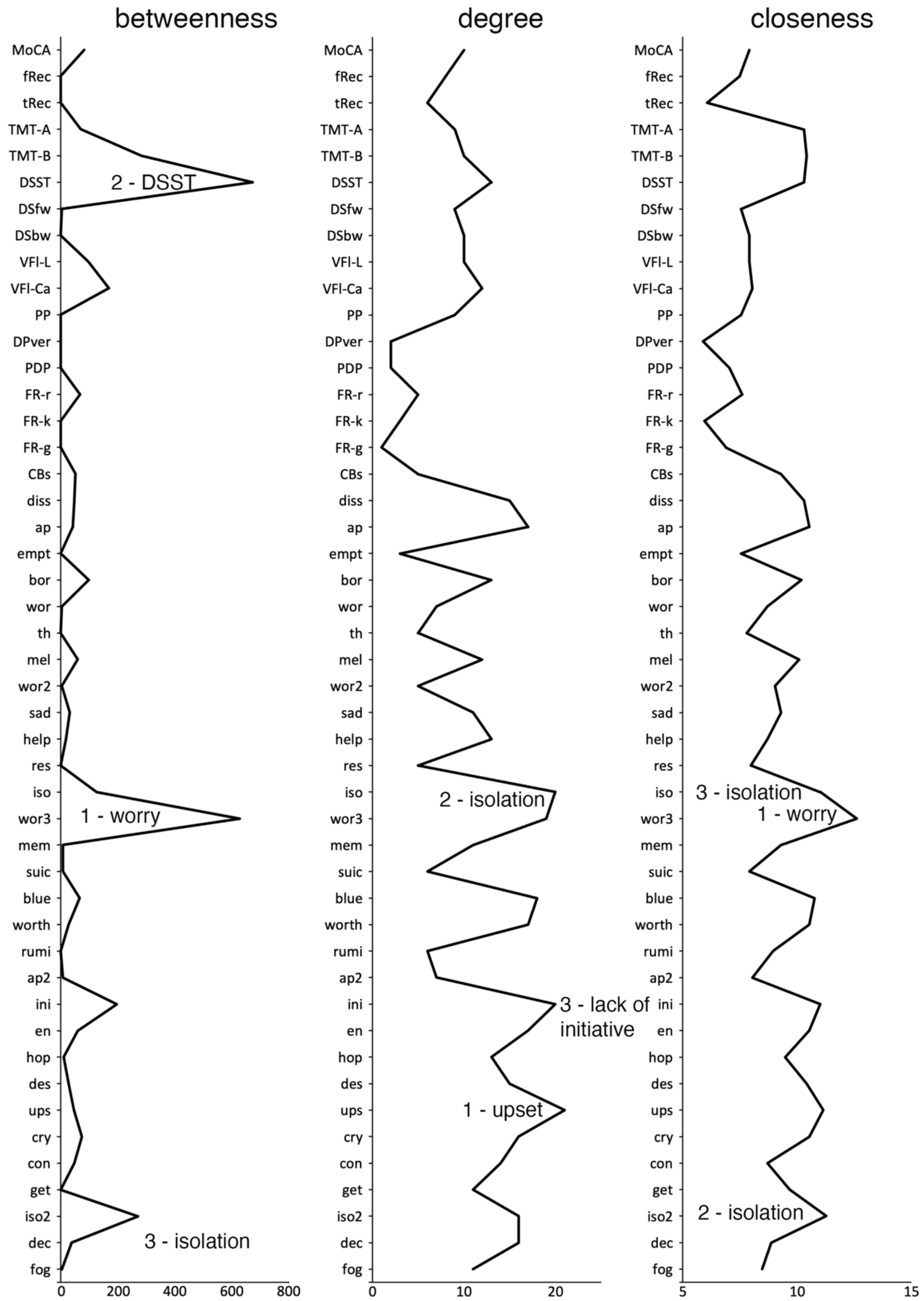

Supplementary Figure 8. Item centrality STRATEGIC.
The relative importance of items in the overall network was quantified by the centrality measures betweenness, degree and closeness. The top three most central items per measure are highlighted. VCI = Vascular Cognitive Impairment. STRATEGIC = White Matter Connections and Memory: The STRATEGIC study. Closeness scores x 10^{-3}. Cognition item DPvis = "doors and people – visual recall", was removed due to weak correlations (r<0.2) with all other network nodes.

**Supplementary Table 12.** STROKDEM: items and abbreviations	
**abbreviation**	**item description**
Cognition	
MoCA	Montreal Cognitive Assessment (total score)
TMT-A	Trail Making Test – part A
TMT-B	Trail Making Test – part B
VFl-Ca	Verbal Fluency – category (animal)
VFl-L	Verbal Fluency – letter
TCFc	Taylor Complex Figure Test – copy
TCFr	Taylor Complex Figure Test – recall
Depression	
bo	CES-D – I was bothered by things that usually don't bother me
app	CES-D – I did not feel like eating; my appetite was poor
blue	CES-D – I felt that I could not shake off the blues even with help from my family or friends
worth	CES-D – I felt I was just as good as other people (worthlessness)
con	CES-D – I had trouble keeping my mind on what I was doing (difficulty concentration)
dep	CES-D – I felt depressed
en	CES-D – I felt that everything I did was an effort (lack of energy)
hop	CES-D – I felt hopeful about the future (hopelessness)
fail	CES-D – I thought my life had been a failure
wor	CES-D – I felt fearful (worry)
in	CES-D – My sleep was restless (insomnia)
sad	CES-D – I was happy (sadness)
talk	CES-D – I talked less than usual
iso	CES-D – I felt lonely (isolation)
unf	CES-D – People were unfriendly
suic	CES-D – I enjoyed life (suicidality)
cry	CES-D – I had crying spells
sad2	CES-D – I felt sad (sadness)
SEs	CES-D – I felt that people disliked me (lack of self-esteem)
ap	CES-D – I could not get going (apathy)

Note. STROKDEM = Study of Factors Influencing Post-Stroke Dementia; CES-D = Centre for Epidemiologic Studies Depression.

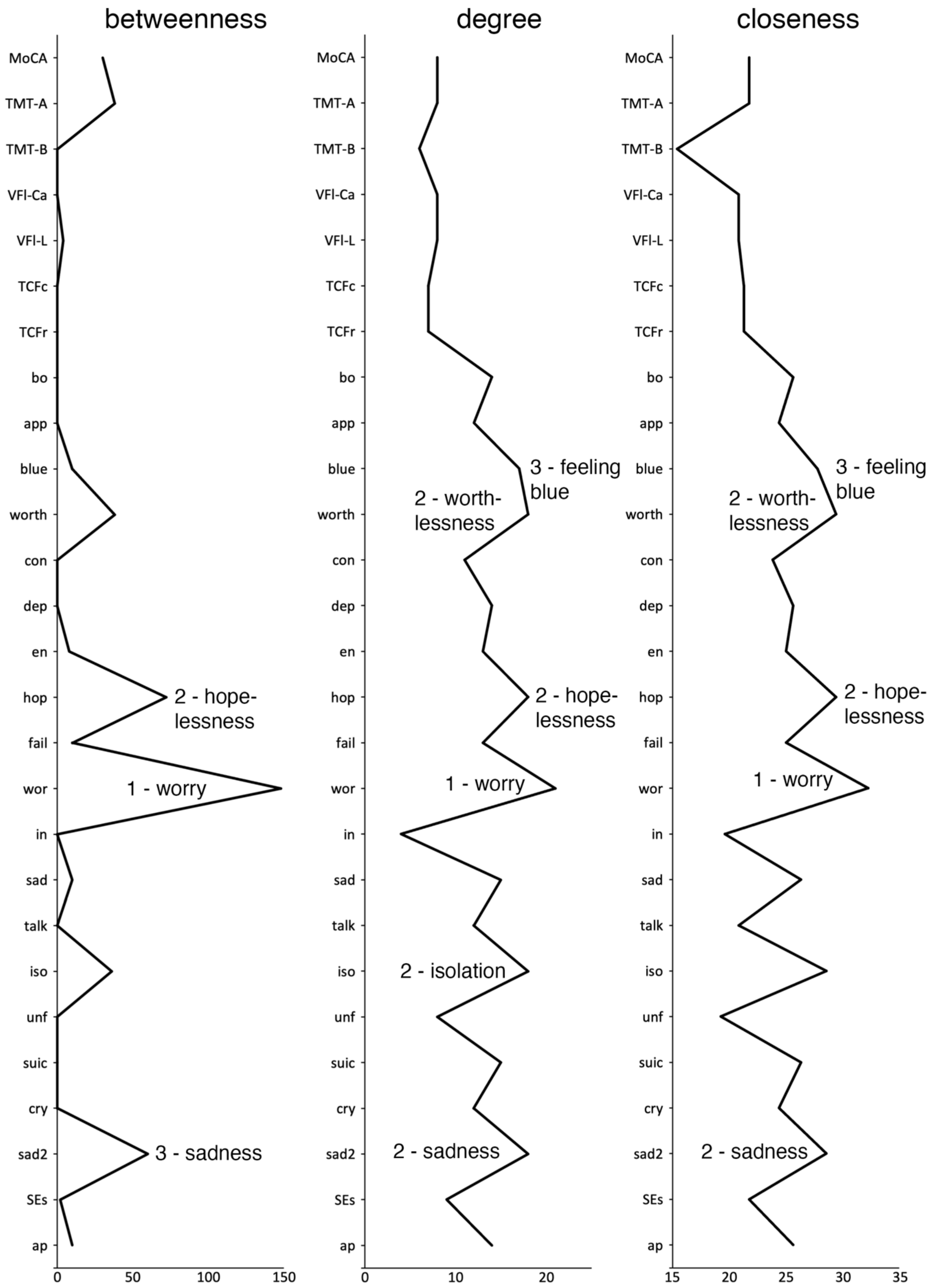

Supplementary Figure 9. Item centrality STOKDEM.
The relative importance of items in the overall network was quantified by the centrality measures betweenness, degree and closeness. The top three most central items per measure are highlighted. VCI = Vascular Cognitive Impairment. STROKDEM = Study of Factors Influencing Post-Stroke Dementia. Closeness scores x 10^{-3}.

**Supplementary Table 13.** Top 3 most central items by study and measure			
study	centrality measure		
	betweenness	degree	closeness
Bulgarian PSS	1. iADL l#<br>2. dissatisfaction l#<br>3. worry *#	1. iADL *#<br>2. hopelessness *#<br>3. worthlessness *#	1. iADL *#<br>2. worthlessness *#<br>3. worry *#
CASPER	1. TMT-A *#<br>2. worry *#<br>3. suicide *#	1. MDD current *#<br>2. suicide *#<br>3. helplessness *#; feeling blue *#; lack of interest *#	1. helplessness *#<br>2. suicide *#<br>3. apathy *#
COAST	1. worry *#<br>2. MoCA *#<br>3. modified Rankin Scale *#	1. MMSE *#; MoCA *#; digit cancellation *#<br>2. FAB *#; verbal fluency (animal) *#; verbal fluency (food) *#; immediate recal *#<br>3. visual reproduction (copy) *#	1. MMSE *#; MoCA *#<br>2. modified Rankin Scale *#<br>3. digit cancellation *#
EpiUSA	1. worry *#<br>2. restlessness *#<br>3. total recall *#	1. worry *#<br>2. dementia *#<br>3. verbal fluency (category) *#; restlessness *#	1. worry *#<br>2. dementia *#<br>3. restlessness *#
Hallym VCI	1. worry *#<br>2. iADL *#<br>3. TMT-B *#	1. iADL *#<br>2. worthlessness *#<br>3. hopelessness *#	1. iADL *#<br>2. worthlessness *#<br>3. hopelessness *#
PROPOLIS	1. apathy *#<br>2. hopelessness *#<br>3. Luria Sequence *#	1. apathy *#<br>2. modified Rankin Scale *#<br>3. Luria Sequence *#; subtraction *#; delayed recall *#; dementia *#	1. apathy *#<br>2. modified Rankin Scale *#<br>3. dementia *#
SSS	1. worry (GDS) *#<br>2. worry (HAM-D) *#<br>3. arithmetic *#	1. worry (GDS) *#<br>2. worry (HAM-D) *#<br>3. MDD + dysthymia *#	1. worry (GDS) *#<br>2. worry (HAM-D) *#<br>3. dissatisfaction *#
STRATEGIC	1. worry *#<br>2. DSST *#<br>3. isolation (avoidance) *#	1. upset *#<br>2. isolation (preference) *#<br>3. lack of initiative *#	1. worry *#<br>2. isolation (preference) *#<br>3. isolation (avoidance) *#
STOKDEM	1. worry *#<br>2. hopelessness *#<br>3. sadness *#	1. worry *#<br>2. worthlessness *#; hopelessness *#; isolation *#; sadness *#<br>3. feeling blue *#	1. worry *#<br>2. worthlessness *#; hopelessness *#; sadness *#<br>3. feeling blue *#
Total frequency across sites	1. 8x: worry<br>2. 3x: iADL or mRS*<br>3. 2x: hopelessness	1. 3x: worry; hopelessness; worthlessness; iADL or mRS*<br>2. 2x: verbal fluency	1. 5x: worry<br>2. 3x: worthlessness; iADL or mRS*<br>3. 2x: hopelessness; dementia;

Note. *activities of daily living item; *depression item; *cognition item; *anxiety item (note that the Hospital Anxiety and Depression Scale used by CASPER is the only questionnaire categorizing worry as an anxiety item instead of a depression item); *other pathology item; *apathy item; #depression module; #cognition module; Bulgarian PSS = Bulgarian Post-Stroke Study; iADL = instrumental

activities of daily living; CASPER = Cognition and Affect after Stroke: Prospective Evaluation of Risks; TMT-A = trail making test – part A; MDD = major depressive disorder; DSST = digit symbol substitution test; COAST = Cognitive Outcome After Stroke; MoCA = Montreal Cognitive Assessment; MMSE = mini mental state examination; FAB = Frontal Assessment Battery; EpiUSA = Epidemiologic Study of the Risk of Dementia After Stroke; Hallym VCI = Hallym Vascular Cognitive Impairment; PROPOLIS = Prospective Study of Pravastatin in the Elderly at Risk; SSS = Sydney Stroke Study; GDS = Geriatric Depression Scale; HAM-D = Hamilton Depression Rating Scale; STRATEGIC = White Matter Connections and Memory: The STRATEGIC study; STROKDEM = Study of Factors Influencing Post-Stroke Dementia. Note that items iADL and mRS are both total scores on scales measuring degree of disability on daily living activities and were therefore summarized as the same item in the total count.

**Supplementary Table 14**. Similarity in betweenness centrality by lesion location across sites				
**Bulgarian PSS**				
	right	left	bilateral	infratentorial
right (n=43)	1	0.48**	N/A	N/A
left (n=35)		1	N/A	N/A
bilateral (N/A)			N/A	N/A
infratentorial (N/A)				N/A
**CASPER**				
	right	left	bilateral	infratentorial
right (n=113)	1	0.23*	N/A	0.23*
left (n=87)		1	N/A	0.29**
bilateral (n=1)[#]			N/A	N/A
infratentorial (n=19)				1
**COAST**				
	right	left	bilateral	infratentorial
right (n=64)	1	0.2	0.31*	0.39**
left (n=39)		1	0.21	0.36*
bilateral (n=26)			1	0.44**
infratentorial (n=9)				1
**EpiUSA**				
	right	left	bilateral	infratentorial
right (n=152)	1	0.37**	N/A	0.28*
left (n=140)		1	N/A	0.21
bilateral (N/A)			N/A	N/A
infratentorial (n=124)				1
**PROPOLIS**				
	right	left	bilateral	infratentorial
right (n=84)	1	0.79***	N/A	0.46**
left (n=107)		1	N/A	0.39*
bilateral (n=0)			N/A	N/A
infratentorial (n=31)				1
**SSS**				
	right	left	bilateral	infratentorial
right (n=19)	1	0.2*	N/A	0.08
left (n=9)		1	N/A	0.21*
bilateral (n=0)			N/A	N/A
infratentorial (n=21)				1
**STRATEGIC**				
	right	left	bilateral	infratentorial
right (n=25)	1	0.19	N/A	N/A
left (n=27)		1	N/A	N/A
bilateral (n=0)			N/A	N/A
infratentorial (n=0)				N/A

STROKDEM				
	right	left	bilateral	infratentorial
right (n=69)	1	0.34*	N/A	N/A
left (n=70)		1	N/A	N/A
bilateral (n=2)[#]			N/A	N/A
infratentorial (N/A)				N/A

Note. *p < 0.05, **p < 0.01, ***p < 0.001, [#]sample size too small to calculate correlation; Bulgarian PSS = Bulgarian Post-Stroke Study; CASPER = Cognition and Affect after Stroke: Prospective Evaluation of Risks; COAST = Cognitive Outcome After Stroke; EpiUSA = Epidemiologic Study of the Risk of Dementia After Stroke; Hallym VCI = Hallym Vascular Cognitive Impairment; PROPOLIS = Prospective Study of Pravastatin in the Elderly at Risk; SSS = Sydney Stroke Study; STRATEGIC = White Matter Connections and Memory: The STRATEGIC study; STROKDEM = Study of Factors Influencing Post-Stroke Dementia.

**Supplementary Table 15.** Similarity in degree centrality by lesion location across sites				
**Bulgarian PSS**				
	right	left	bilateral	infratentorial
right (n=43)	1	0.5**	N/A	N/A
left (n=35)		1	N/A	N/A
bilateral (N/A)			N/A	N/A
infratentorial (N/A)				N/A
**CASPER**				
	right	left	bilateral	infratentorial
right (n=113)	1	0.46***	N/A	0.21*
left (n=87)		1	N/A	0.42***
bilateral (n=1)[#]			N/A	N/A
infratentorial (n=19)				1
**COAST**				
	right	left	bilateral	infratentorial
right (n=64)	1	0.64***	0.63***	0.54***
left (n=39)		1	0.57***	0.56***
bilateral (n=26)			1	0.5***
infratentorial (n=9)				1
**EpiUSA**				
	right	left	bilateral	infratentorial
right (n=152)	1	0.88***	N/A	0.8***
left (n=140)		1	N/A	0.79***
bilateral (N/A)			N/A	N/A
infratentorial (n=124)				1
**PROPOLIS**				
	right	left	bilateral	infratentorial
right (n=84)	1	0.76***	N/A	0.32
left (n=107)		1	N/A	0.47**
bilateral (n=0)			N/A	N/A
infratentorial (n=31)				1

SSS				
	right	left	bilateral	infratentorial
right (n=19)	1	0.47***	N/A	0.35***
left (n=9)		1	N/A	0.3***
bilateral (n=0)			N/A	N/A
infratentorial (n=21)				1
**STRATEGIC**				
	right	left	bilateral	infratentorial
right (n=25)	1	0.32*	N/A	N/A
left (n=27)		1	N/A	N/A
bilateral (n=0)			N/A	N/A
infratentorial (n=0)				N/A
**STROKDEM**				
	right	left	bilateral	infratentorial
right (n=69)	1	0.92***	N/A	N/A
left (n=70)		1	N/A	N/A
bilateral (n=2)[#]			N/A	N/A
infratentorial (N/A)				N/A

Note. *p < 0.05, **p < 0.01, ***p < 0.001, [#]sample size too small to calculate correlation; Bulgarian PSS = Bulgarian Post-Stroke Study; CASPER = Cognition and Affect after Stroke: Prospective Evaluation of Risks; COAST = Cognitive Outcome After Stroke; EpiUSA = Epidemiologic Study of the Risk of Dementia After Stroke; Hallym VCI = Hallym Vascular Cognitive Impairment; PROPOLIS = Prospective Study of Pravastatin in the Elderly at Risk; SSS = Sydney Stroke Study; STRATEGIC = White Matter Connections and Memory: The STRATEGIC study; STROKDEM = Study of Factors Influencing Post-Stroke Dementia.

Supplementary Table 16. Similarity in closeness centrality by lesion location across sites

Bulgarian PSS				
	right	left	bilateral	infratentorial
right (n=43)	1	0.6**	N/A	N/A
left (n=35)		1	N/A	N/A
bilateral (N/A)			N/A	N/A
infratentorial (N/A)				N/A
**CASPER**				
	right	left	bilateral	infratentorial
right (n=113)	1	0.5***	N/A	0.21*
left (n=87)		1	N/A	0.54***
bilateral (n=1)[#]			N/A	N/A
infratentorial (n=19)				1
**COAST**				
	right	left	bilateral	infratentorial
right (n=64)	1	0.69***	0.66***	0.42**
left (n=39)		1	0.43**	0.43**
bilateral (n=26)			1	0.47***
infratentorial (n=9)				1

EpiUSA				
	right	left	bilateral	infratentorial
right (n=152)	1	0.88***	N/A	0.63***
left (n=140)		1	N/A	0.69***
bilateral (N/A)			N/A	N/A
infratentorial (n=124)				1
**PROPOLIS**				
	right	left	bilateral	infratentorial
right (n=84)	1	0.90***	N/A	0.33*
left (n=107)		1	N/A	0.43**
bilateral (n=0)			N/A	N/A
infratentorial (n=31)				1
**SSS**				
	right	left	bilateral	infratentorial
right (n=19)	1	0.49***	N/A	0.37***
left (n=9)		1	N/A	0.2*
bilateral (n=0)			N/A	N/A
infratentorial (n=21)				1
**STRATEGIC**				
	right	left	bilateral	infratentorial
right (n=25)	1	0.29*	N/A	N/A
left (n=27)		1	N/A	N/A
bilateral (n=0)			N/A	N/A
infratentorial (n=0)				N/A
**STROKDEM**				
	right	left	bilateral	infratentorial
right (n=69)	1	0.74***	N/A	N/A
left (n=70)		1	N/A	N/A
bilateral (n=2)#			N/A	N/A
infratentorial (N/A)				N/A

Note. *p < 0.05, **p < 0.01, ***p < 0.001, #sample size too small to calculate correlation; Bulgarian PSS = Bulgarian Post-Stroke Study; CASPER = Cognition and Affect after Stroke: Prospective Evaluation of Risks; COAST = Cognitive Outcome After Stroke; EpiUSA = Epidemiologic Study of the Risk of Dementia After Stroke; Hallym VCI = Hallym Vascular Cognitive Impairment; PROPOLIS = Prospective Study of Pravastatin in the Elderly at Risk; SSS = Sydney Stroke Study; STRATEGIC = White Matter Connections and Memory: The STRATEGIC study; STROKDEM = Study of Factors Influencing Post-Stroke Dementia.

Node label abbreviations and item centrality measures by depression scale

**Supplementary Table 17.** GDS: included items and abbreviations	
**abbreviation**	**item description**
Cognition	
zAtt	standardized cognitive domain score attention & processing speed
zMem	standardized cognitive domain score memory
zLan	standardized cognitive domain score language
zExec	standardized cognitive domain score frontal executive
zMot	standardized cognitive domain score perceptual motor
Depression	
diss	GDS15 – Are you basically satisfied with your life? (dissatisfied)
ap	GDS15 – Have you dropped many of your activities and interests? (apathy)
empt	GDS15 – Do you feel that your life is empty?
bor	GDS15 – Do you often get bored?
mel	GDS15 – Are you in good spirits most of the time? (melancholia)
wor	GDS15 – Are you afraid that something bad is going to happen to you? (worry)
sad	GDS15 – Do you feel happy most of the time? (sadness)
help	GDS15 – Do you often feel helpless?
iso	GDS15 – Do you prefer to stay at home rather than go out and do things? (isolation)
mem	GDS15 – Do you feel you have more problems with memory than most?
suic	GDS15 – Do you think it is wonderful to be alive now? (suicidality)
worth	GDS15 – Do you feel pretty worthless the way you are now?
en	GDS15 – Do you feel full of energy? (lack of energy)
hop	GDS15 – Do you feel that your situation is hopeless?
des	GDS15 – Do you think that most people are better off than you are? (destitute)

Note. GDS15 = 15-item Geriatric Depression Scale.

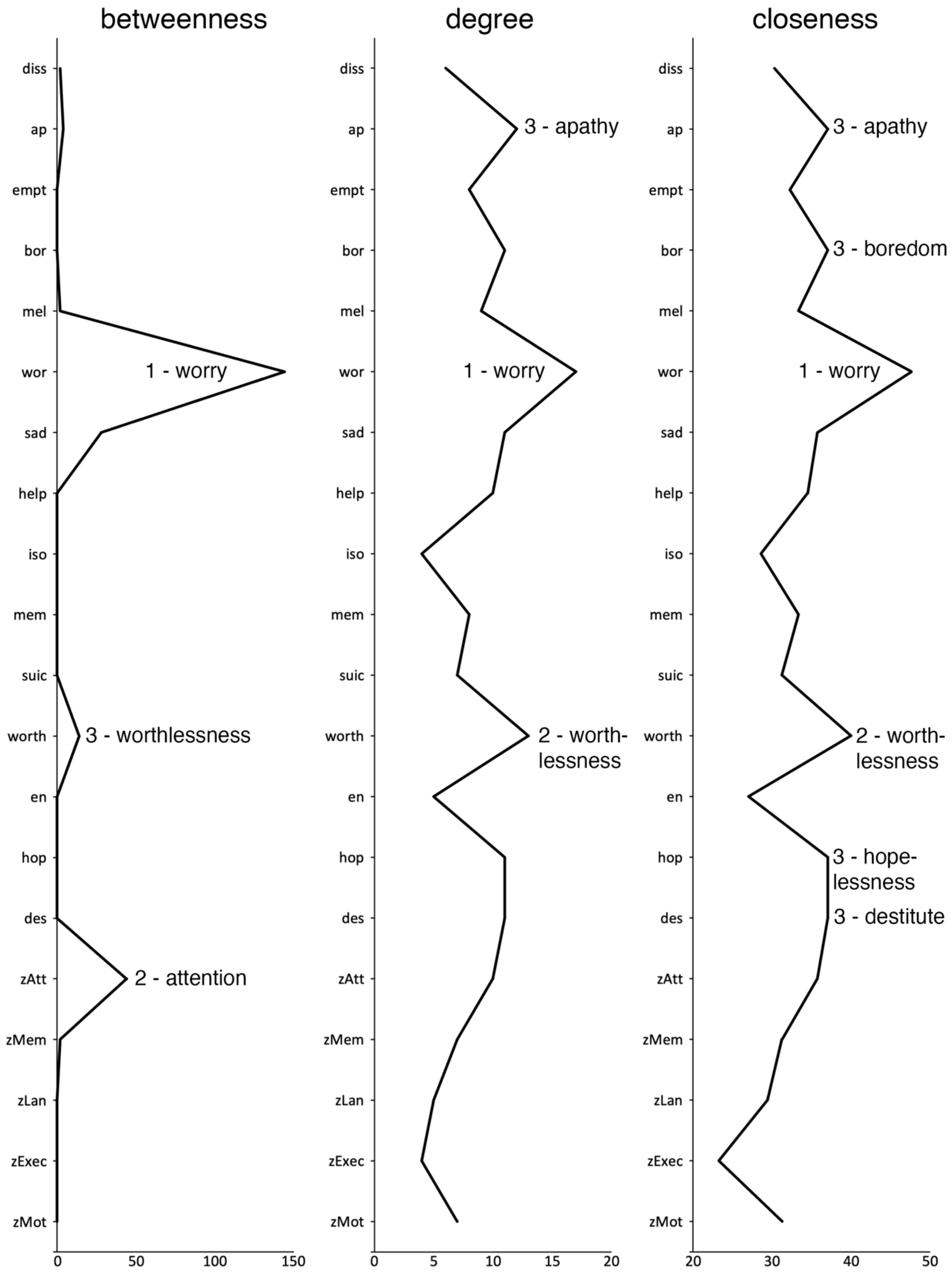

Supplementary Figure 10. Item centrality GDS.
The relative importance of items in the overall network was quantified by the centrality measures betweenness, degree and closeness. The top three most central items per measure are highlighted. GDS = Geriatric Depression Scale. Closeness scores x 10^{-3}.

**Supplementary Table 18.** HAM-D: included items and abbreviations	
**abbreviation**	**item description**
Cognition	
zAtt	standardized cognitive domain score attention & processing speed
zMem	standardized cognitive domain score memory
zLan	standardized cognitive domain score language
zExec	standardized cognitive domain score frontal executive
zMot	standardized cognitive domain score perceptual motor
Depression	
blue	HAM-D – Depressed mood (gloomy attitude, pessimism about the future, feeling of sadness, tendency to weep) (blue)
glt	HAM-D – Feelings of guilt
suic	HAM-D – suicide
in(i)	HAM-D – insomnia initial (difficulty falling asleep)
in(m)	HAM-D – insomnia middle (Complains of being restless and disturbed during the night. Waking during the night)
in(d)	HAM-D – insomnia delayed (Waking in early hours of the morning and unable to fall asleep again)
ap	HAM-D – work and interests (Slowness of thought, speech, and activity; apathy; stupor)
res	HAM-D – retardation (Restlessness associated with anxiety)
agi	HAM-D – agitation
wor	HAM-D – anxiety psychic (worry)
som	HAM-D – anxiety somatic (Gastrointestinal, indigestion, Cardiovascular, palpitation, Headaches, Respiratory, Genito-urinary, etc.)
app	HAM-D – somatic symptoms: gastrointestinal (Loss of appetite, heavy feeling in abdomen; constipation)
en	HAM-D – somatic symptoms: general (Heaviness in limbs, back or head; diffuse backache; loss of energy and fatiguability) (lack of energy)
lib	HAM-D – genital symptoms (Loss of libido, menstrual disturbances)
hyp	HAM-D – hypochondriasis
wei	HAM-D – weight loss
ins	HAM-D – insight (Insight must be interpreted in terms of patient's understanding and background.) (lack of insight)

Note. HAM-D = Hamilton Depression Rating Scale.

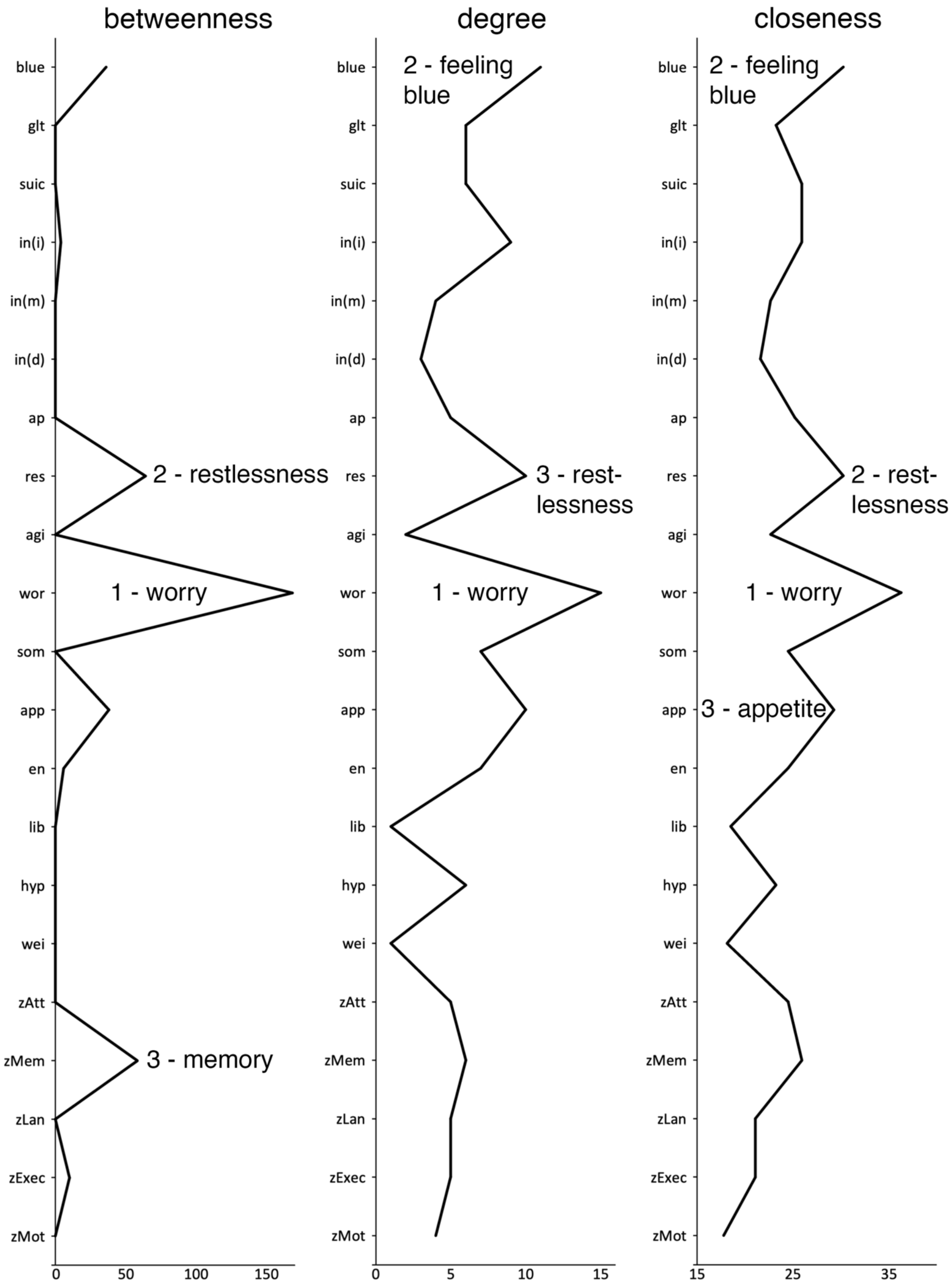

Supplementary Figure 11. Item centrality HAM-D.
The relative importance of items in the overall network was quantified by the centrality measures betweenness, degree and closeness. The top three most central items per measure are highlighted. HAM-D = Hamilton Depression Rating Scale. Closeness scores x 10^{-3}. The depression item ins = "lack of insight" was removed due to weak correlations (r<0.2) with all other network nodes.

Centrality measures: Exploratory follow-up analysis of worry

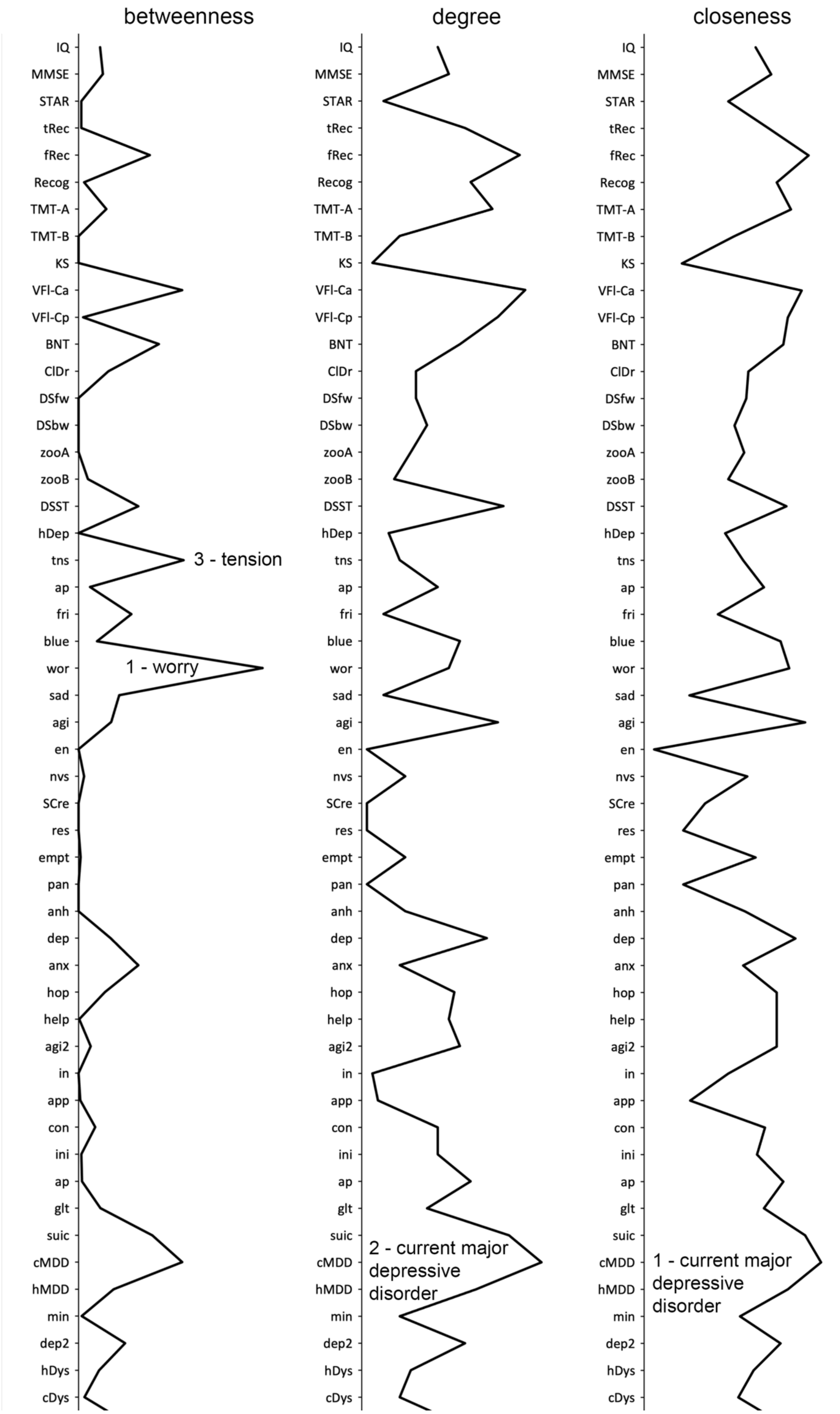

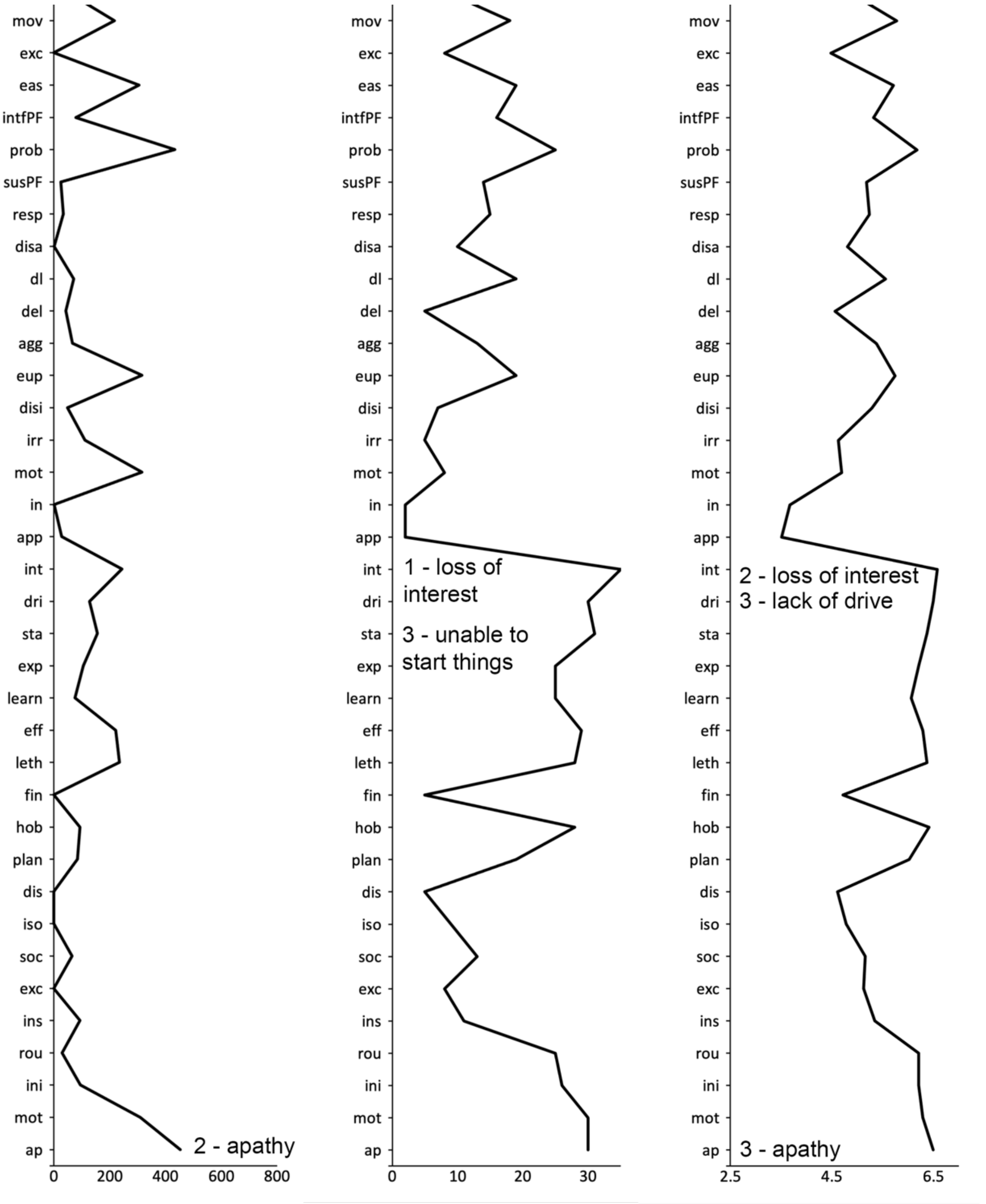

Supplementary Figure 12. Item centrality CASPER worry+ group.
The relative importance of items in the overall network was quantified by the centrality measures betweenness, degree and closeness. The top three most central items per measure are highlighted. CASPER = Cognition and Affect after Stroke: Prospective Evaluation of Risks. Closeness scores x 10^{-3}. The psychopathology item euph = “euphoria” was removed due to weak correlations (r<0.2) with all other network nodes.

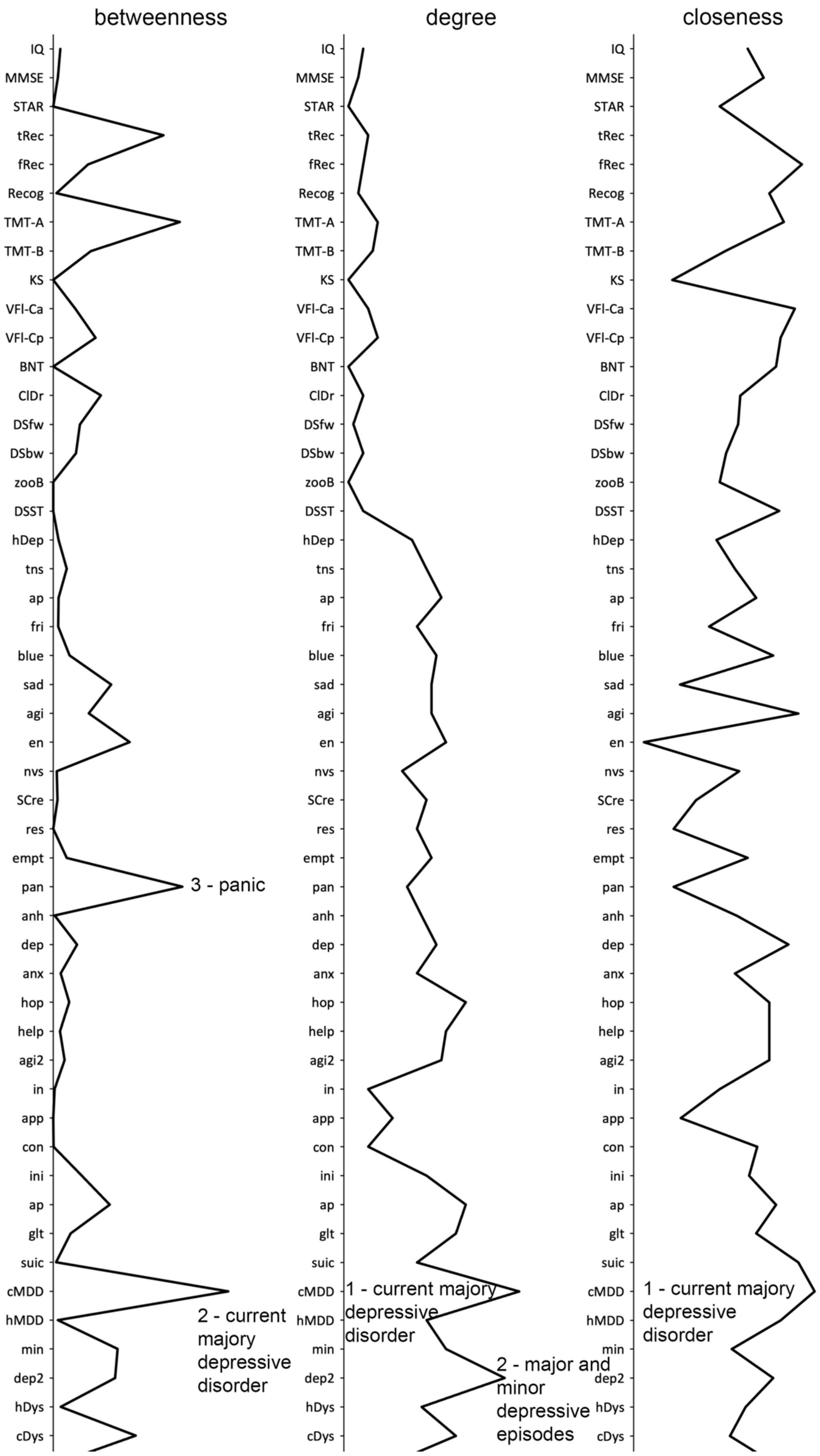
betweenness
degree
closeness
IQ
MMSE
STAR
tRec
fRec
Recog
TMT-A
TMT-B
KS
VFl-Ca
VFl-Cp
BNT
ClDr
DSfw
DSbw
zooB
DSST
hDep
tns
ap
fri
blue
sad
agi
en
nvs
SCre
res
empt
pan
anh
dep
anx
hop
help
agi2
in
app
con
ini
ap
glt
suic
cMDD
hMDD
min
dep2
hDys
cDys
3 - panic
2 - current majory depressive disorder
1 - current majory depressive disorder
2 - major and minor depressive episodes
1 - current majory depressive disorder

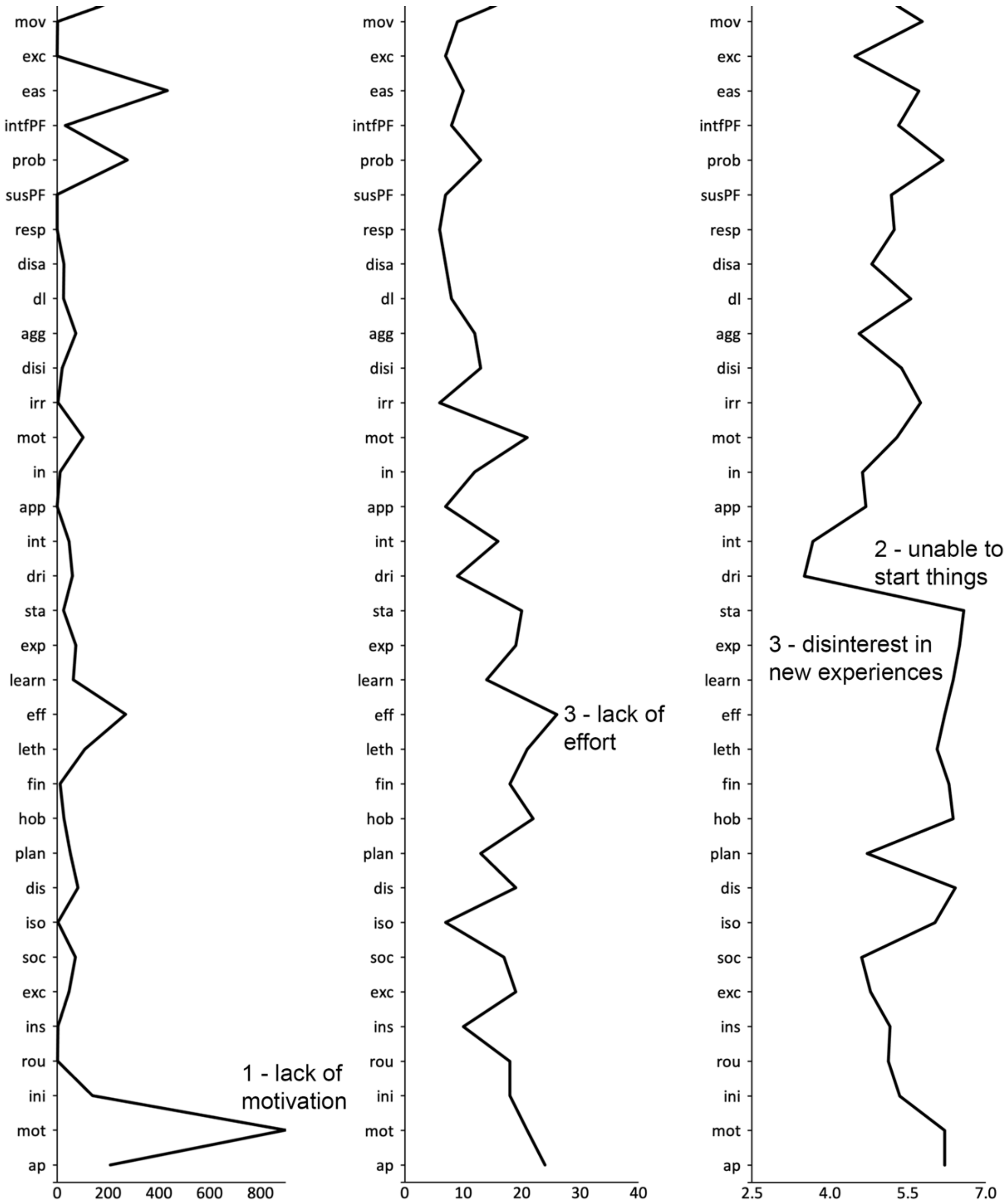

Supplementary Figure 13. Item centrality CASPER worry- group.
The relative importance of items in the overall network was quantified by the centrality measures betweenness, degree and closeness. The top three most central items per measure are highlighted. CASPER = Cognition and Affect after Stroke: Prospective Evaluation of Risks. Closeness scores x 10^{-3}. The cognition item zooA = "zoo A", the depression item wor = "worry" and the psychopathology items del = "delusions", hall = "hallucinations" and euph = "euphoria" were removed due to weak correlations (r<0.2) with all other network nodes.

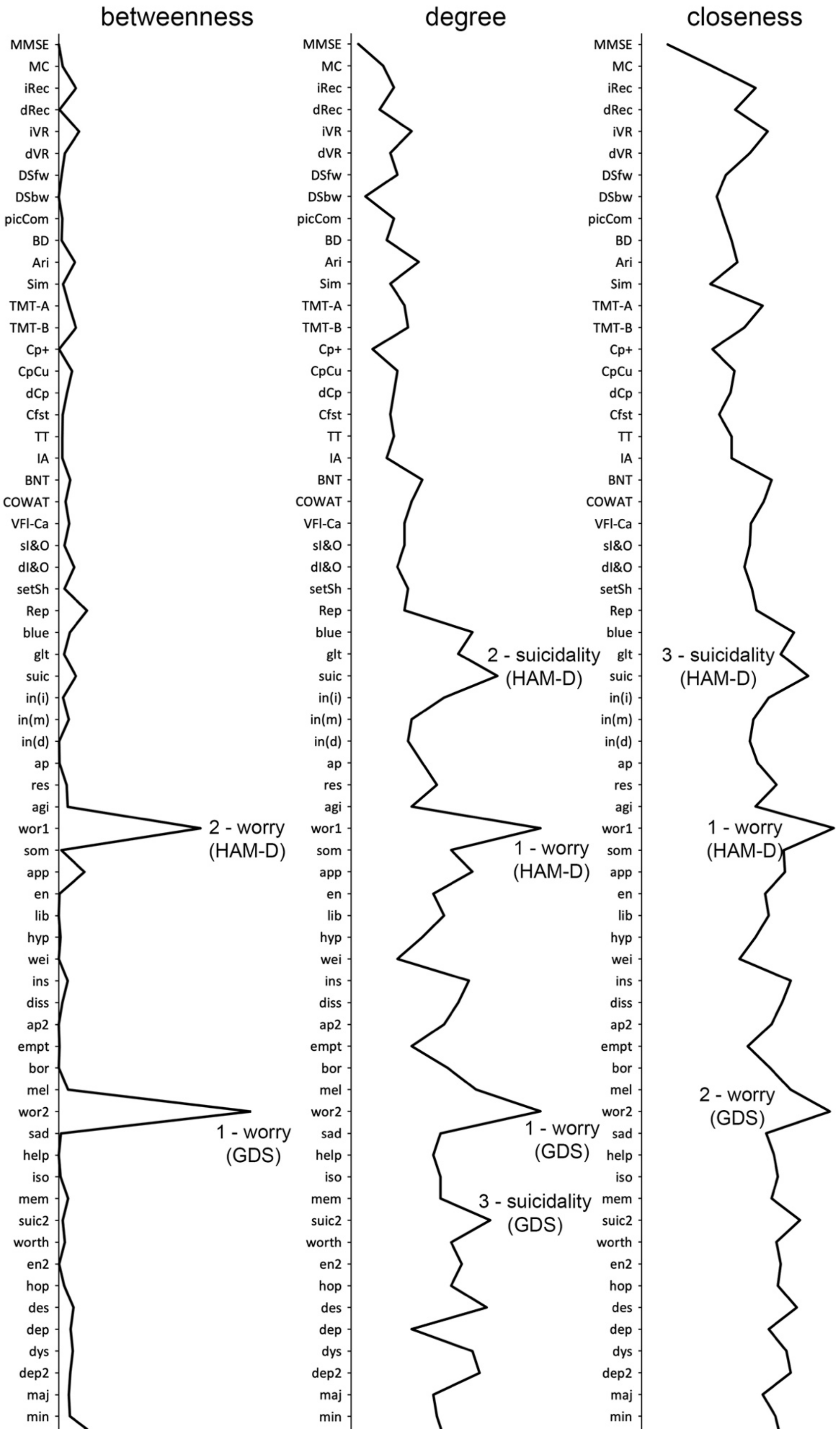
betweenness
degree
closeness
MMSE
MC
iRec
dRec
iVR
dVR
DSfw
DSbw
picCom
BD
Ari
Sim
TMT-A
TMT-B
Cp+
CpCu
dCp
Cfst
TT
IA
BNT
COWAT
VFl-Ca
sI&O
dI&O
setSh
Rep
blue
glt
suic
in(i)
in(m)
in(d)
ap
res
agi
wor1
som
app
en
lib
hyp
wei
ins
diss
ap2
empt
bor
mel
wor2
sad
help
iso
mem
suic2
worth
en2
hop
des
dep
dys
dep2
maj
min
2 - worry (HAM-D)
1 - worry (GDS)
2 - suicidality (HAM-D)
1 - worry (HAM-D)
1 - worry (GDS)
3 - suicidality (GDS)
3 - suicidality (HAM-D)
1 - worry (HAM-D)
2 - worry (GDS)

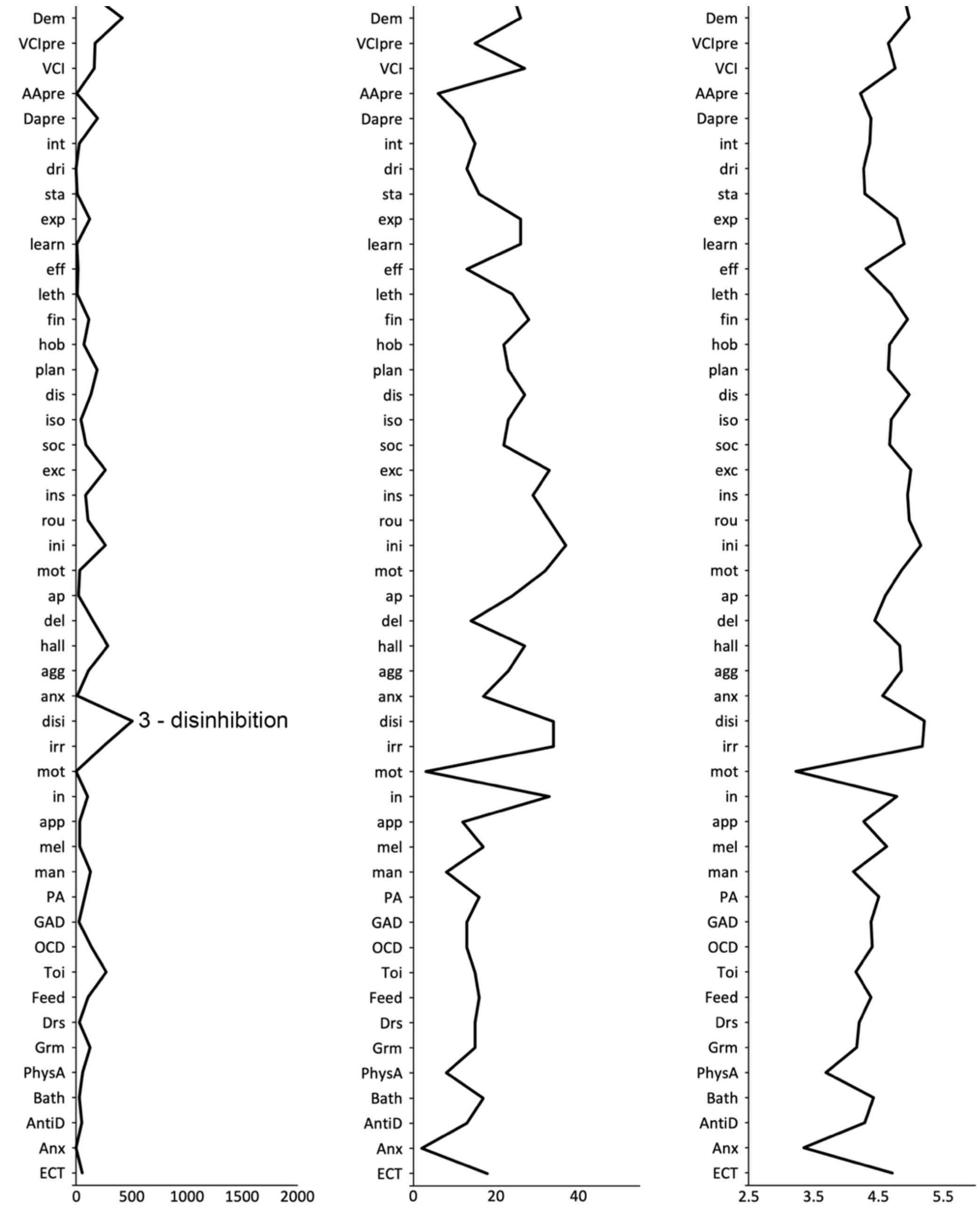

Supplementary Figure 14. Item centrality SSS worry+ group.
The relative importance of items in the overall network was quantified by the centrality measures betweenness, degree and closeness. The top three most central items per measure are highlighted. SSS = Sydney Stroke Study. Closeness scores x 10^{-3}. Psychopathology items euph = “euphoria”, SZ = “schizophrenia”, SZfm = “schizophreniform disorder”, delD = “delusional disorder”, bPD = “brief psychotic disorder”, mPD = “medication induced psychotic disorder”, sPsy = “substance induced psychotic disorder”, oPsy = “psychotic disorder not otherwise specified”, mix = “mood disorder with mixed features”, hyMan = “hypomania”, adjD = “adjustment disorder”, Ap = “agoraphobia”, PaDAp+ = “panic disorder with agoraphobia”, PaDAp- = “panic disorder without agoraphobia” and the medication item AntiP = “Antipsychotics” were removed due to weak correlations (r<0.2) with all other network nodes.

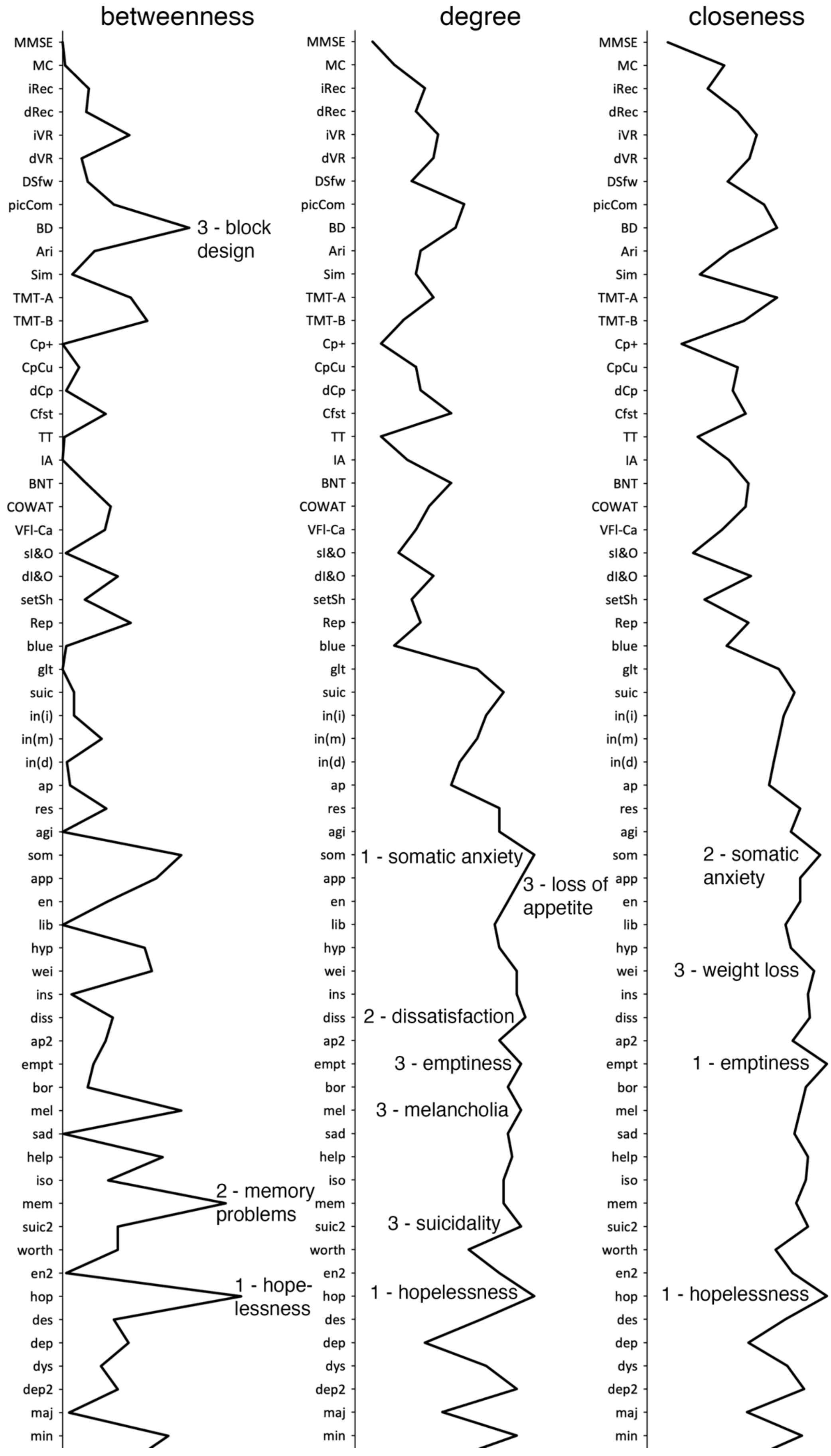
betweenness
degree
closeness
MMSE
MC
iRec
dRec
iVR
dVR
DSfw
picCom
BD
Ari
Sim
TMT-A
TMT-B
Cp+
CpCu
dCp
Cfst
TT
IA
BNT
COWAT
VFl-Ca
sI&O
dI&O
setSh
Rep
blue
glt
suic
in(i)
in(m)
in(d)
ap
res
agi
som
app
en
lib
hyp
wei
ins
diss
ap2
empt
bor
mel
sad
help
iso
mem
suic2
worth
en2
hop
des
dep
dys
dep2
maj
min
3 - block design
2 - memory problems
1 - hopelessness
1 - somatic anxiety
3 - loss of appetite
2 - dissatisfaction
3 - emptiness
3 - melancholia
3 - suicidality
1 - hopelessness
2 - somatic anxiety
3 - weight loss
1 - emptiness
1 - hopelessness

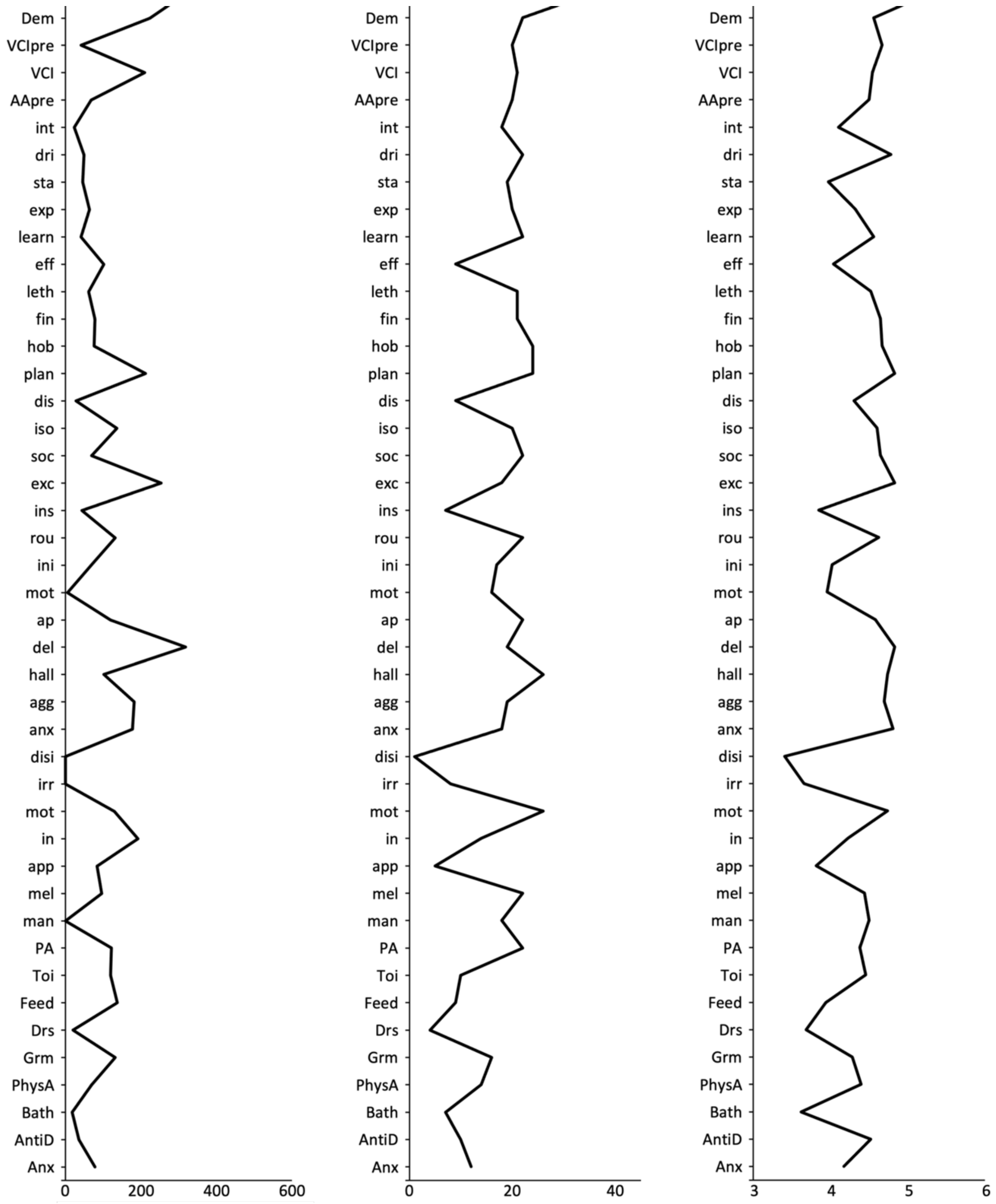

Supplementary Figure 15. Item centrality SSS worry- group.
The relative importance of items in the overall network was quantified by the centrality measures betweenness, degree and closeness. The top three most central items per measure are highlighted. SSS = Sydney Stroke Study. Closeness scores x 10^{-3}. The cognitive item DSbw = "Digit Span – backward", the depression item wor = "worry" (GDS and HAM-D items), psychopathology items euph = "euphoria", SZ = "schizophrenia", SZfm = "schizophreniform disorder", delD = "delusional disorder", bPD = "brief psychotic disorder", mPD = "medication induced psychotic disorder", sPsy = "substance induced psychotic disorder", oPsy = "psychotic disorder not otherwise specified", mix = "mood disorder with mixed features", hyMan = "hypomania", adjD = "adjustment disorder", Ap = "agoraphobia", PaDAp+ = "panic disorder with agoraphobia", PaDAp- = "panic disorder without agoraphobia", GAD = "generalized anxiety disorder", OCD = "obsessive compulsive disorder" and the pathology and medication items AntiP = "Antipsychotics", ECT = "electroconvulsive therapy" and DApre = "drug abuse prior to stroke" were removed due to weak correlations (r<0.2) with all other network nodes.
